# Preharvest Methyl Jasmonate Treatment Affects the Mineral Profile, Metabolites, and Antioxidant Capacity of Radish Microgreens Produced without Substrate

**DOI:** 10.3390/foods13050789

**Published:** 2024-03-04

**Authors:** Shimeles Tilahun, Min Woo Baek, Ki-Seok An, Han Ryul Choi, Jong Hwan Lee, Su Ho Tae, Do Su Park, Jin Sung Hong, Cheon Soon Jeong

**Affiliations:** 1 Agriculture and Life Science Research Institute, Kangwon National University, Chuncheon 24341, Republic of Korea; shimeles@kangwon.ac.kr (S.T.); parkds@kangwon.ac.kr (D.S.P.); 2 Department of Horticulture and Plant Sciences, Jimma University, Jimma 378, Ethiopia; 3 Interdisciplinary Program in Smart Agriculture, Kangwon National University, Chuncheon 24341, Republic of Korea; minwoo100@kangwon.ac.kr (M.W.B.); ljhhh96@kangwon.ac.kr (J.H.L.); xotngh9906@kangwon.ac.kr (S.H.T.); 4 Department of Horticulture, Kangwon National University, Chuncheon 24341, Republic of Korea; tjdrhdgkwk36@kangwon.ac.kr; 5 Eco-Friendly Agricultural Product Safety Center, Kangwon National University, Chuncheon 24341, Republic of Korea; 6 National Institute of Horticultural and Herbal Science, Rural Development Administration, Wanju-gun 55365, Republic of Korea; hanryul192@kangwon.ac.kr; 7 Department of Applied Biology, Kangwon National University, Chuncheon 24341, Republic of Korea

**Keywords:** amino acids, antioxidant capacity, glucosinolates, MeJA, secondary metabolites, microgreens, radish

## Abstract

This study investigated the impact of Methyl Jasmonate (MeJA) application on the nutritional content and yield of five different colored radish microgreens. Microgreens were produced without substrate and subjected to 0.5 mM and 1.0 mM MeJA treatments on the 7th day, three days before harvest. The parameters measured included yield, dry matter, minerals, amino acids, secondary metabolites such as chlorophylls (Chls), anthocyanins, flavonoids, phenolics, glucosinolates (GSLs), vitamin C, and antioxidant capacity. MeJA at 1.0 mM generally improved yield and dry weight across cultivars, and all microgreens exhibited rich mineral and amino acid composition, with the influence of cultivar being more significant than MeJA treatment. However, MeJA enhanced all cultivars’ anthocyanins, GSLs, phenolics, flavonoids, and antioxidant activities. Generally, as the antioxidant capacity is the primary factor influencing the nutritional quality of microgreens, MeJA-treated microgreens, especially with selected superior cultivars such as ‘Asia purple’ and ‘Koregon red’, could offer a potential for cultivation of value-added, eco-friendly microgreens with substrate-free cultivation.

## 1. Introduction

Vegetables of the Brassicaceae family have a global distribution and are found on every continent except Antarctica [1]. They are known for their rich content of health-promoting secondary metabolites, including phenolic compounds, glucosinolates, natural pigments, and vitamins [2,3]. The content and composition of antioxidants and their antioxidant activity exhibit variations between and within subspecies of the Brassicaceae family [3] and within the radish cultivars themselves [4]. Radish cultivars exhibited variations in their phytochemical content across seeds, sprouts at varying stages, and mature taproots [4]. Comparative studies assessing the nutrient content between radish microgreens and their mature counterparts found that microgreens exhibited higher nutritional levels compared to their mature parts [5,6].

Microgreens have attracted attention due to their short growing cycle, efficient utilization of space, vibrant colors, diverse flavors, and rich phytonutrients [7,8]. Microgreens and sprouts from the Brassicaceae family, particularly radish (*Raphanus sativus* L.), contain significant amounts of antioxidants, vitamin C, and health-promoting compounds such as glucosinolates (GSLs) and phenolic compounds and have been shown to have antioxidant and anticancer properties, both in vivo and in vitro [7,9].

Plants have developed various defense mechanisms and adaptive responses to survive and thrive in challenging conditions [10]. Plant hormones act as key regulators in plant responses to both abiotic and biotic stresses [11]. Methyl Jasmonate (MeJA), the derivative of jasmonic acid (JA), is among these plant hormones that are involved in regulating adverse environmental conditions through JA signaling pathways [2,11]. When specific parts of the plant experience localized damage (wound), the plant activates JA-dependent transcriptional reprogramming throughout the plant parts due to the movement of signaling molecules across cells, tissues, and organs [12]. JA can be transmitted systemically either through the plant’s vascular bundles or by airborne means [11] for long-distance transmission of a danger signal to initiate plant defenses. In contrast to JA, which cannot easily penetrate the cell membrane, MeJA can be transmitted via air and penetrate the cell membrane due to its high volatility [11]. The JA-induced plant’s defense mechanisms primarily involve the activation of antioxidative enzymes and bioactive phytochemical compounds [13,14], which enhances the medicinal and nutritional values of plants [9]. Therefore, factors affecting phytochemical content and profile in the production of Brassicaceae plants are valuable considerations for both plant and human health [14].

Sakamoto and Suzuki [15] reported an increase in total phenolic content and radical scavenging capacity by the co-treatment of MeJA and salinity. In addition, MeJA has been found to increase the anthocyanin content of sprouts grown in light [16]. Anthocyanin-rich radish microgreens have different colors that can draw consumers’ attention. In addition to providing colors to plant tissues, anthocyanins also play a crucial role in a range of plant physiological processes, such as protection against UV irradiation, attracting pollinators, and scavenging reactive oxygen species [17]. Zhang et al. [17] reported a positive correlation between the increase in antioxidant capacity of radish microgreens and their content of anthocyanins.

A vertical multilayered growing system stands out as the best solution for sustaining the continuously growing global population. This method remains unaffected by adverse weather conditions, ensuring year-round production while maximizing space efficiency and minimizing water usage [18]. Using a vertical growing system for sprouting has led to a fourfold increase in radish microgreen yield per square meter compared to conventional growing system [19].

Generally, the quality of vegetables is influenced by cultivar selection, growing systems, and treatments with exogenous elicitors such as MeJA. In this research, we investigated the impact of MeJA treatment at 0.5 mM and 1.0 mM concentrations on five radish microgreen cultivars grown in a multilayered vertical growing unit to identify whether the improvement of their secondary phytochemicals is achievable without compromising yield. MeJA is an approved safe compound when applied before harvest, and treatment concentrations were based on the ranges of previous recommendations for improving the quality of different *Brassica* vegetables [18,20] and our preliminary tests on radish microgreens. We hypothesized that MeJA treatment, particularly at 1.0 mM, could be useful for improving bioactive phytochemicals (secondary metabolites) as a defense mechanism in response to the stress signal from the sprayed MeJA, which in turn may improve nutritional metabolites and antioxidant capacity. For this purpose, the study examined the fresh weight, dry matter, and contents of minerals, amino acids, Chls, GSLs, anthocyanins, flavonoids, phenolics, and antioxidant capacity of the control and MeJA-treated radish microgreens grown on trays by sprouting method in a multilayered growth unit.

## 2. Materials and Methods

### 2.1. Plant Material

The seeds of five distinct radish cultivars that are commonly distributed in Korea for sprout production and displaying varied shoot colorations (namely, ‘Asia purple’, ‘Asia green 1’, ‘Asia green 2’, ‘Koregon red’, ‘Asia red’, and ‘Asia purple’) were sourced from Asia Seed Co., LTD, Seoul, Korea. Since radishes are known to generate tall shoots, radish microgreens were grown using a ‘sprouting’ technique. The trays for growing were purchased as a set, which included a cover with perforations for air circulation, a seed tray (mesh tray), and a water holder (lower tray). The size dimensions of the set were 20 cm in width, 30 cm in length, and 15 cm in height (including the cover) (Figure S1). To enhance germination, 10 g of seeds from each cultivar were soaked in tap water for 24 h. After 24 h, the seeds were evenly scattered across the surface of the seed trays, with a 10 g per tray seeding density. After uniform distribution of the seeds on the seed tray, 2 L of water were added to the lower tray, reaching the level of the seed holder mesh tray. This setup facilitates rapid germination by allowing the seeds to rest on the water holder tray, ensuring a continuous water supply as the roots begin to grow down. The mean weight of 1000 seeds for ‘Asia purple’, ‘Koregon red’, ‘Asia red’, ‘Asia green 1’, and ‘Asia green 2’ were recorded as 16.39, 17.23, 10.22, 10.63, and 16.21 g, respectively (Table S1). The germination rate for ‘Asia green 2’ and ‘Asia purple’ cultivars was determined to be 99%, whereas the ‘Asia red’, ‘Asia green 1’, and ‘Koregon red’ cultivars displayed a germination rate of 98% (Table S1). In this study, the seeds were sown on a soilless tray with mesh designed to suspend the roots in clean, fresh water. The water was changed daily until harvesting to prevent contamination.

The trays were set up at Kangwon National University (37°52′ N, 127°44′ E; the Republic of Korea) in a vertical multilayered growing unit utilizing a completely randomized design with three replications. The growing room comprised multiple growing units spaced 0.5 m apart from each other and the wall. Each growing unit consisted of a 1.0 × 0.5 m shelf with four layers, providing the capacity to accommodate six growing trays per layer. For our study, a total of 45 growing trays were used, distributing three trays for each combination of cultivar and treatment. These trays were arranged on two growing units in dark conditions for 2 days. After the initial 2-day period, the sprouts were exposed to the light cycle comprising a 16 h light period with a photosynthetic flux density (PPFD) of 600 μmol m^−2^s^−1^ and 6500 K cool daylight spectrum, followed by an 8 h dark period. The temperature was kept at 20 ± 2 °C over the whole growth period until they reached the stage of fully developed cotyledons.

After the microgreens grew well and attained a stage of 7 days after sowing, an aqueous solution of 0.5 mM and 1.0 mM MeJA (Sigma-Aldrich, St. Louis, MO, USA) in 0.1% ethanol was sprayed on all parts of the shoots above the mesh to saturation, with a 50 mL test solution per tray three days prior to harvest, and the control plants were applied with a 0.1% ethanol solution [18,20]. Treatment concentrations were based on the ranges of previous recommendations for different *Brassica* vegetables [18,20] and our preliminary tests on radish microgreens. Therefore, the microgreens of the five radish cultivars were subjected to three different treatments: treatment A (control), treatment B (0.5 mM MeJA), and treatment C (1.0 mM MeJA). A total of 45 trays were used in the study, five cultivars with the three treatments in three replications (Table S2).

### 2.2. Fresh Weight and Dry Matter

The harvesting stage of all cultivars was attained on the 10th day after sowing (Figure S1). The microgreens were harvested by cutting the seedlings above the seed tray’s mesh and weighed to record their fresh weight. Subsequently, the samples were sorted and subjected to drying in a forced drought oven at 65 °C to assess the dry matter. For analysis, three replicates of the remaining samples underwent freeze drying using a vacuum freeze drier (FDT-8650, Operon, Gimpo-si, South Korea), and the dried samples were subsequently ground to powder. After being ground into powder, the microgreen samples were sieved through 40 µm mesh, placed in LDPE pouches, and kept at −20 °C until they were needed for analysis.

### 2.3. Mineral Contents

Samples of freeze-dried microgreens of the tested radish cultivars were submitted to the National Instrumentation Center for Environmental Management (NICEM) Laboratory. Samples were analyzed for total nitrogen using the Kjeldahl method [21] and macrominerals (P, K, Ca, Mg, and Na) and microminerals (Mn, Fe, Cu, Mo, and Zn) were tested following microwave-assisted acid digestion standard method 3051 [22] using inductively coupled plasma optical emission spectroscopy (5800 ICP-OES, Agilent, Santa Clara, CA, USA).

### 2.4. Measurement of Amino Acids

For each treatment, three replicates of freeze-dried microgreens were extracted by 75% ethanol, followed by ultrasonic and room-temperature extraction for 1 h and 24 h, respectively. After filtering the extract with a 0.2 µL filter, analysis of amino acid content was conducted by Dionex Ultimate 3000 HPLC (Conquer Scientific, Poway, CA, USA) using the method described by [23].

### 2.5. Secondary Metabolites

#### 2.5.1. Chlorophylls

Chls were extracted from the ethanol extract of each cultivar. The DMSO (dimethyl sulfoxide) chlorophyll extraction procedure was used as described by [18]. The absorbance readings were measured at 645 nm and 663 nm using a microplate reader (SpectraMax ABS Plus, Molecular Devices, Sunnyvale, CA, USA) against a DMSO blank. Following that, Chl *a*, Chl *b*, and total Chls were calculated using the equations developed by Arnon [24], as outlined below:

- Chl *a* (mg g^−1^ dry weight) = [(12.7 × A663) − (2.69 × A645)] × (V/1000 × W)
- Chl *b* (mg g^−1^ dry weight) = [(22.9 × A645) − (4.68 × A663)] × (V/1000 × W)
- Total Chls = Chl *a* + Chl *b*

V represents the volume of solvent and W corresponds to the dry weight of the extracted tissue

#### 2.5.2. Anthocyanin Content

Total anthocyanin (cyanidin-3-glucoside equivalents) content was analyzed using the pH differential method [25]. Freeze-dried radish microgreen samples of each treatment (0.25 g) were combined with 5 mL of methanol containing 0.1% HCl. Subsequently, the mixed samples were subjected to ultrasonic treatment three times for 10 min, and the resulting extract was centrifuged to separate the supernatant. A 50 μL portion of the supernatant was acquired using a 0.45 μm membrane filter (PTFE, 13 mm, Whatman, Maidstone, UK). Additionally, a 25 mM potassium chloride buffer (pH 1.0) and 400 mM sodium acetate buffer (pH 4.5) were mixed in 950 μL, respectively, and the mixture was developed for 15 min. The readings were made at 520 nm and 700 nm using a microplate reader (SpectraMax ABS Plus, Molecular Devices, Sunnyvale, CA, USA).

$$\mathrm{Anthocyanin}\;(\mathrm{mg}\;g^{-1})=\frac{V\times A\times MW\times DF}{\varepsilon \times m}$$

where the variables V, A, MW, DF, ε, and m represent the total volume of extract (mL), the difference between the absorbance values at pH 1.0 and pH 4.5 under 520 nm and 700 nm, molar mass of cyanidin-3-glucoside (449.2 g mol^−1^), dilution factor, molar extinction coefficient in L mol^−1^ cm^−1^ (26,900), and sample quantity (g), respectively.

#### 2.5.3. Vitamin C Content

Three freeze-dried microgreen samples of each treatment weighing 1.0 g were mixed with 10 mL of 5% meta-phosphoric acid and homogenized for 1 min. The homogenized sample was centrifuged (14,000× *g* for 10 min) and the liquid layer of extracts was membrane-filtered (0.22 µm) and analyzed by HPLC as described by [26] using a ZORBAX Eclipse XDB-C18 (4.6 × 250 mm, 5 µm, Agilent, Santa Clara, CA, USA) column and detector (UV-2075, Jasco, Tokyo, Japan) at 265 nm. The mobile phase (methanol to 0.1 M KH_2_PO_4_ in 1:9 ratio) was injected at a volume of 20 µL and flowed at a rate of 1 mL min^−1^.

#### 2.5.4. Total Phenolics and Flavonoids

The total phenolic and flavonoid contents of freeze-dried radish microgreen samples from each treatment were determined by microplate reader (Spectramax i3, Molecular Devices, Sunnyvale, CA, USA) using a methodology previously implemented in our laboratory and described by Tilahun et al. [27].

#### 2.5.5. Glucosinolates Analysis

The desulfo-GSLs were extracted from 200 mg sample in accordance with Ku et al. [28]. The methods by Han et al. [29] were used for the separation and quantification of desulfo-GSLs using a Dionex UltiMate 3000 ultra-high-performance liquid chromatography (UHPLC) system that was equipped with a column oven, pump, auto-sampler, and diode array detector (all Thermo Fisher Scientific, Waltham, MA, USA).

### 2.6. Antioxidant Capacity

Freeze-dried and ground radish microgreen samples of each treatment were extracted using the methodology described by Baek et al. [18], which had previously been implemented in our laboratory. The antioxidant capacity in three (DPPH, ABTS, and FRAP) assays were performed in triplicate at three sample concentrations (1.0, 2.5, and 5.0 mg L^−1^), according to Baek et al. [18] and expressed as Trolox-equivalent antioxidant capacity (TEAC) in μmol TE for each sample concentration [30].

### 2.7. Experimental Design and Statistical Analysis

A completely randomized design was employed for the experiment. The data obtained were subjected to analysis of variance (ANOVA) to assess the differences between treatments at *p* < 0.05 by statistical software (SAS/STAT ^®^ 9.1; SAS Institute Inc., Cary, NC, USA). To further examine the variations between the treatments, Duncan’s multiple range test was conducted. To visualize the differences between treatments, heat map and principal component analysis (PCA) were made by MetaboAnalyst v5.0 and XLSTAT version 2015.1 (Addinsoft Inc., 244 Fifth Avenue, Suite E100, New York, NY, USA). Additionally, the collected parameters were correlated using Pearson’s correlation test.

## 3. Results and Discussion

The microgreens of five radish cultivars (‘Koregon red’, ‘Asia purple’, ‘Asia green 1’, ‘Asia green 2’, and ‘Asia red’) grown with a vertical multilayered system without substrate were subjected to three different treatments: control, 0.5 mM MeJA, and 1.0 mM MeJA. Analysis of variance (ANOVA) was conducted to assess the significant differences among the five radish microgreen cultivars and three treatments on fresh weight, dry matter, mineral contents, amino acids, secondary metabolites (Chls, anthocyanins, vitamin C, total phenolics, total flavonoids, GSLs), and antioxidant capacity with three different assays. The results revealed significant variations in the collected parameters attributed to the different cultivars and treatments.

### 3.1. Fresh Weight, Dry Matter and Mineral Contents

**Figure 1:**
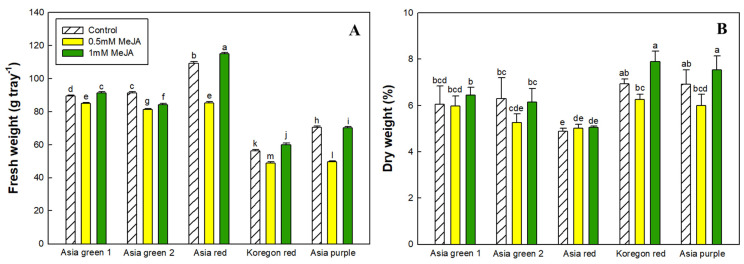
Fresh weight per tray (**A**) and percentage dry weight (**B**) of five radish microgreen cultivars, cultivated without substrate and harvested on the 10th day, influenced by the interaction between the cultivars and preharvest MeJA treatment at 0.5 mM and 1.0 mM concentrations applied on the 7th day post-sowing. Vertical bars indicate three replicates’ mean values ± standard error. Different letters on the bars indicate significant interaction differences between cultivars and treatments with Duncan’s mean separation at α = 0.05.

Based on our preliminary tests, the microgreens were harvested at the optimum stage (on the 10th day) because early harvesting negatively impacts the yield, while delayed harvesting compromises the quality, as the growth is entirely based on the stored food reserve of the seed. The food stored in the seed is sufficient to attain the cotyledon stage for most vegetable microgreens [31]. Following the harvest on the 10th day, fresh weight, dry matter, and mineral content measurements were taken to assess the impact of MeJA concentrations on the radish microgreens. The interaction results demonstrated the significant influence of MeJA treatment on the fresh and dry weight and mineral contents of the five radish microgreen cultivars (Figure 1 and Table 1). In the control group, the fresh weight measurements ranged from 56.17 g tray^−1^ in ‘Koregon red’ to 109.20 g tray^−1^ in ‘Asia red’. In the treatment group, the average fresh yields for 0.5 mM MeJA-treated microgreens were 84.97, 81.17, 85.27, 48.90, and 49.73 g tray^−1^ for ‘Asia green 1’, ‘Asia green 2’, ‘Asia red’, ‘Koregon red’, and ‘Asia purple’, respectively. Alternatively, the average fresh yields for 1.0 mM MeJA-treated microgreens were 91.23, 84.23, 115.10, 60.03, and 70.10 g tray^−1^ for ‘Asia green 1’, ‘Asia green 2’, ‘Asia red’, ‘Koregon red’, and ‘Asia purple’, respectively (Figure 1A). The results revealed that application of 0.5 mM MeJA significantly reduced the fresh weight when compared to the control group. On the other hand, the treatment with 1.0 mM MeJA led to an increase in yield, except in the case of ‘Asia green 2’, where the fresh weight was lower than the control group but still higher than that observed with 0.5 mM MeJA. Consistent with our results, Singh et al. [31] documented yields of 95.56 and 94.89 g fresh weight per tray from 9.05 and 10.73 g seeds in white and pink radish cultivars, respectively. These were achieved through cultivation on a substrate composed of coco peat, vermiculite, and perlite mixture in a 5:2:1 ratio, with harvesting taking place on the 10th day after sowing. Utilizing the vertical microgreen cultivation system employed in our study, it becomes possible to make efficient use of space, with four layers at a convenient human height, resulting in four times the space utilization compared to traditional surface cultivation.

Similarly, the results of dry weight percentage in the control group ranged from 4.88% in ‘Asia red’ to 6.94% in ‘Koregon red’. Upon the application of 0.5 mM MeJA treatment to the microgreens, the dry weight percentages were 5.97, 5.25, 5.01, 6.25, and 5.99% for ‘Asia green 1’, ‘Asia green 2’, ‘Asia red’, ‘Koregon red’, and ‘Asia purple’, respectively. On the other hand, the dry weight percentages for 1.0 mM MeJA-treated microgreens were 6.44, 6.15, 5.04, 7.89, and 7.53% for ‘Asia green 1’, ‘Asia green 2’, ‘Asia red’, ‘Koregon red’, and ‘Asia purple’, respectively (Figure 1B). The results agree with the findings of Wojdyło et al. [32]. The high water content in ‘Asia red’ could be attributed to their advanced vegetative growth stage and their ability to store water effectively, as well as their adaptations to minimize water loss [32].

These findings indicate that application of 1.0 mM MeJA generally led to increased fresh yields and dry weight percentage across most cultivars compared to both the control group and the 0.5 mM MeJA treatment. However, the response to MeJA treatments varied among the different cultivars, indicating cultivar-specific effects on fresh weight and dry weight percentage under MeJA exposure.

Table 1 reveals significant variations in concentrations of macro- and micro-elements, which were influenced by the interactions between different radish microgreen cultivars and treatments. Among the macronutrients, N, P, and K were the most abundant, accounting for 65.84%, 12.99%, and 10.02% of the total minerals’ concentration, respectively. Following these, Ca, Mg, Na, Fe, Zn, Mn, Cu, and Mo constituted smaller proportions, making up approximately 5.18%, 4.37%, 1.43%, 0.08%, 0.04%, 0.03%, 0.003%, and 0.001% of the total minerals’ concentration, respectively. In the control group, N was found to be the predominant element in all cultivars, with concentrations ranging from 46.84 g kg^−1^ in ‘Asia red’ to 65.68 g kg^−1^ DW in ‘Koregon red’, making up an average of 67.87%, 62.40%, 61.84%, 70.43%, and 64.91% of the total minerals’ concentration in ‘Asia green 1’, ‘Asia green 2’, ‘Asia red’, ‘Koregon red’, and ‘Asia purple’, respectively. A similar trend was observed in MeJA-treated microgreens, where N remained the most abundant element, with concentrations ranging from 64.18% in ‘Asia purple’ to 67.73% in ‘Asia green 1’, at 0.5 mM concentration and from 63.01% in ‘Asia green 2’ to 68.37% in ‘Asia green 1’ at 1.0 mM concentration, respectively.

Vegetables can be an important source of nitrate (NO_3_^−^) due to the use of agricultural fertilizers, and NO_3_^−^ can accumulate in plants and could cause health issues [33]. However, in our study we deliberately excluded the use of fertilizer and substrate, thereby eliminating the presence of NO_3_^−^ in the microgreens. By adopting the sprouting method and relying solely on water and seeds without any agricultural inputs, our approach offers microgreens with reduced NO_3_^−^ content. As a result, the consumption of these microgreens could help lower the intake of nitrate associated with vegetable consumption.

The average P concentration in radish microgreens was measured at 10.70 g kg^−1^ DW, ranging from 9.46 g kg^−1^ DW in the control ‘Asia green 1’ to 11.79 g kg^−1^ DW in 1.0 mM MeJA-treated ‘Asia green 2’. Following P, K content averaged 8.25 g kg^−1^ DW, ranging from 6.65 g kg^−1^ DW in 1.0 mM MeJA-treated ‘Asia purple’ to 10.37 g kg^−1^ DW in the control ‘Asia red’. Ca was the fourth most abundant macro-element, with an average concentration of 4.28 g kg^−1^ DW, varying from 3.54 g kg^−1^ DW in the control ‘Asia green 2’ to 5.39 g kg^−1^ DW in 0.5 mM MeJA-treated ‘Asia purple’. Mg content ranged from 3.40 g kg^−1^ DW in the control ‘Koregon red’ to 3.87 g kg^−1^ DW in 1.0 mM MeJA-treated ‘Asia green 2’, with an average of 3.60 g kg^−1^ DW across all cultivars and treatments. Considering the recommended daily intake of P (700 mg), K (2700–4700 mg), Ca (800 mg), and Mg (350 mg) [34,35], incorporating radish microgreens into the daily diet could be a beneficial contribution to meeting these dietary requirements.

In addition, the average Na content in radish microgreens was measured at 1.18 g kg^−1^ DW, with a range of 0.91 in 0.5 mM MeJA-treated ‘Asia green 2’ to 1.46 g kg^−1^ DW in 1.0 mM MeJA-treated ‘Koregon red’. Na is known as an antinutrient, but the findings from this study indicate that radish microgreens contain relatively low levels of Na. Consequently, these microgreens could be an excellent choice for individuals who need to follow low-sodium diets.

Micromineral analysis revealed that Fe was the most abundant, followed by Zn, Mn, Cu, and Mo, with an average content of 69.50, 31.90, 26.16, 2.53, and 0.52 mg kg^−1^ DW, respectively, across all cultivars and treatments (Table 1). Considering the recommended dietary allowance for these microminerals, incorporating radish microgreens into a daily diet could contribute to fulfilling the requirements for Fe (8–18 mg per day for adults and 27 mg per day for pregnant women), Zn (2–5 mg for infants and pre-school children and up to 8–11 mg per day for adult women and men, respectively), Mn (1.8–2.3 mg per day for adult females and males), Cu (1 mg per day for adult women and men), and Mo (45 μg per day for adult women and men) [34,35]. Overall, the effects of MeJA treatments on mineral contents varied among the different cultivars of radish microgreens, suggesting that there are cultivar-specific responses to MeJA exposure. Despite these differences, incorporating radish microgreens into a balanced diet, regardless of the treatments, provides a natural and beneficial way to supplement essential minerals for overall health and well-being.

### 3.2. Amino Acids

Amino acids play essential roles in a wide array of biochemical processes, encompassing protein synthesis, cell signaling, osmoregulation, and metabolic regulation, among others [36]. Their role in these diverse physiological functions underscores the fundamental importance of amino acids in maintaining the intricate balance and functionality of living organisms. This study identified 22 amino acids that exhibited significant differences among the five cultivars of radish microgreens, with the total free amino acids reduced significantly by MeJA treatment (Table 2). Glutamine emerged as the most abundant amino acid, followed by histidine and asparagine, across all five tested cultivars. The impact of MeJA concentration on the total free amino acids is highly dependent on the individual cultivar. The contents of total free amino acids were higher in the control groups of ‘Koregon red’, followed by ‘Asia purple’ and ‘Asia red’, respectively. In the control group, the total free amino acids ranged from 52.55 g kg^−1^ DW in ‘Asia green 2’ to 69.66 g kg^−1^ DW in ‘Koregon red’. However, with the application of MeJA, total free amino acids ranged from 43.73 g kg^−1^ DW in ‘Asia green 2’ to 59.60 g kg^−1^ DW in ‘Asia purple’, and from 44.67 g kg^−1^ DW in ‘Asia green 2’ to 60.89 g kg^−1^ DW in ‘Koregon red’ at 0.5 mM and 1.0 mM concentrations, respectively (Table 2 and Figure S2). One of the ways MeJA promotes plant defense is by stimulating the production of secondary metabolites and the reduction of amino acids in response to MeJA treatment, which could be due to trade-offs and costs associated with defense mechanisms [37].

The quality of protein in a food can be evaluated by analyzing the levels of nine essential amino acids (EAA). These amino acids are histidine, isoleucine, leucine, lysine, methionine, phenylalanine, threonine, tryptophan, and valine [38]. Assessing the presence and concentrations of these EAA provides valuable insights into the nutritional value and protein quality of the food under study. From the results of this study, MeJA treatment at both concentrations either maintained or reduced the total EAAs, except in ‘Asia purple’ and ‘Asia green 2’, where 0.5 mM and 1.0 mM concentrations increased the total EAAs, respectively. Generally, the total EAAs ranged from 11.93 g kg^−1^ DW in ‘Asia green 2’ treated with 1.0 mM MeJA to 18.39 g kg^−1^ DW in ‘Asia purple’ treated with 0.5 mM MeJA (Table 2 and Figure S2). γ-aminobutyric acid (GABA) has received significant attention for its essential roles as an inhibitory neurotransmitter within the central nervous system, and this neurotransmitter plays a crucial role in reducing blood pressure, inducing relaxation, and even contributing to improvements in the body’s immune response [39]. In this study, GABA contents were also reduced in response to MeJA application at both 0.5 mM and 1.0 mM concentrations, except in the case of ‘Asia red’ at 0.5 mM MeJA and ‘Asia purple’ at both concentrations, where GABA contents were maintained and increased, respectively. The GABA contents ranged from 108.60 mg kg^−1^ DW in ‘Asia red’ treated with 1.0 mM MeJA to 262.06 mg kg^−1^ DW in ‘Asia purple’ treated with 0.5 mM MeJA (Table 2 and Figure S2). Similarly, the changes in proline, an amino acid known for its significance in plants under stress conditions [40], did not exhibit consistent patterns with the application of MeJA. The inconsistent pattern in the contents of both GABA and proline could be due to short time exposure of microgreens to MeJA as it was applied on the 7th day and harvesting was done on the 10th day. These findings suggest that the impact of MeJA on amino acid accumulation is highly dependent on the individual characteristics of each cultivar, and the choice of cultivars could be more important than the MeJA treatment to produce radish microgreens rich in total and high-quality amino acids.

### 3.3. Secondary Metabolites

#### 3.3.1. Chlorophylls and Anthocyanins

**Figure 2:**
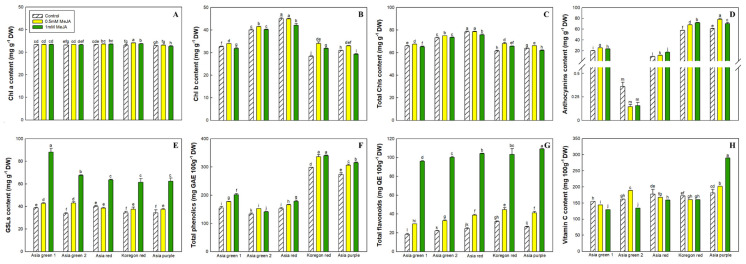
The chlorophyll a (**A**), chlorophyll b (**B**), total chlorophylls (**C**), anthocyanins (**D**), total glucosinolates (**E**), total phenolics (**F**), total flavonoids (**G**), and vitamin C (**H**) contents of five radish microgreen cultivars, cultivated without substrate and harvested on the 10th day, influenced by the interaction between the cultivars and preharvest MeJA treatment at 0.5 mM and 1.0 mM concentration applied on the 7th day post-sowing. Vertical bars indicate three replicates’ mean values ± standard error. Different letters on the bars indicate significant interaction differences between cultivars and treatments with Duncan’s mean separation at α = 0.05. Chl and GSLs stand for chlorophyll and glucosinolates, respectively.

Vegetables are rich in various natural bioactive compounds, such as pigments (Chls and carotenoids), minerals, vitamins, and antioxidants [39]. Beyond their role in photosynthesis, pigments play a crucial part in influencing consumers’ preferences by indicating maturity, quality, and freshness [41]. Moreover, Chls demonstrate significant antioxidant properties [42], while dietary anthocyanin consumption has been associated with reduced risk factors for cardiovascular diseases [43]. Hence, the content of Chls and anthocyanins content could serve as quality indicators in radish microgreens. The results of our study showed a significant effect of MeJA treatment on Chls content for all the five cultivars of microgreens. MeJA treatment at 0.5 mM either maintained (for ‘Asia green 1’ and ‘Asia purple’) or increased (for the other three cultivars) Chl *a* content (Figure 2A). Similarly, MeJA treatment at 0.5 mM either maintained (for ‘Asia red’) or increased (for the other four cultivars) the contents of Chl *b* and total Chls (Figure 2B,C). However, the effect of MeJA treatment at 1.0 mM had no consistent trend and showed cultivar dependent effects. For instance, the application of MeJA at 1.0 mM reduced the contents of Chl *b* and total Chls for ‘Asia green 1’, ‘Asia red’, and ‘Asia purple’, while it maintained Chl *b* and total Chls for ‘Asia green 2’ and increased for ‘Koregon red’ (Figure 2B,C). Generally, the average total Chls ranged from 61.94 mg g^−1^ DW in ‘Asia purple’ treated with MeJA at 1.0 mM to 79.57 mg g^−1^ DW in ‘Asia red’ treated with MeJA at 0.5 mM (Figure 2C). The study demonstrated that MeJA at 0.5 mM significantly improved the total Chls in four of the tested cultivars and maintained it in one cultivar.

On the other hand, the application of MeJA treatment had a significant effect of increasing the anthocyanin content of the tested microgreens, except for ‘Asia green 2’, where no significant changes were observed at both 0.5 mM and 1.0 mM concentrations. Nevertheless, it is crucial to note that the relationship between the MeJA concentration and anthocyanins content did not follow a linear trend (Figure 2D). The findings highlight that MeJA at both 0.5 mM and 1.0 mM concentrations resulted in a significant improvement in the anthocyanins content across the four tested cultivars. Notably, there was wide variation among cultivars in the anthocyanin content that ranged from 0.13 mg g^−1^ DW in ‘Asia green 2’ treated with 0.5 mM MeJA to 78.11 mg g^−1^ DW in ‘Asia purple’ treated with 0.5 MeJA (Figure 2D).

#### 3.3.2. Glucosinolates

As shown in Figure 2E, there were lower levels of total GSLs in all tested cultivars in the control and 0.5 mM MeJA-treated microgreens compared to the1.0 mM MeJA-treated microgreens. In the absence of MeJA treatment, the total GSLs ranged from 33.54 mg g^−1^ DW in ‘Asia green 2’ to 39.80 mg g^−1^ DW in ‘Asia red’. MeJA treatment significantly increased the total GSLs content of all the tested cultivars; the changes are high, especially at 1.0 mM concentrations. The application of 1.0 mM MeJA led to an increase in the total GSLs across all tested cultivars, ranging from 54.86 mg g^−1^ DW in ‘Asia red’ to 88.22 mg g^−1^ DW in ‘Asia green 1’. Specifically, the 1.0 mM MeJA treatment led to 2.3-, 1.9-, 1.4-, 1.7-, and 1.7-fold increases in total GSLs in ‘Asia green 1’, ‘Asia green 2’, ‘Asia red’, ‘Koregon red’, and ‘Asia purple’, respectively, when compared to the control (Figure 2E and Table 3). In this study, a total of eight glucosinolates (glucoiberin, glucoraphanin, gluconapin, hydroxy glucobrassicin, glucoerucin, glucoraphasatin, glucobrassicin, and 4-methoxy glucobrassicin) were identified in both the treated and control microgreens of the five radish cultivars. Among these, glucoraphastin emerged as the most abundant GSL, constituting more than half of the total GSLs in both the control and treatment groups across all cultivars, followed by glucoraphanin (Table 3). Consistent with our results, Demir et al. [44] identified glucoraphasatin and glucoraphanin as the predominant glucosinolates in radish microgreens. Notably, treatment with 1.0 mM MeJA significantly increased glucoraphastin and glucoiberin contents in all the tested cultivars. This observation highlights the potential of 1.0 mM MeJA treatment to enhance the accumulation of these specific GSLs. A previous study on chives showed the dose-dependent effects of preharvest MeJA treatment on secondary metabolites [9], and 0.5 mM MeJA had the most significant effect, specifically on phenolic compounds of substrate-grown chives.

#### 3.3.3. Total Phenolics and Flavonoids Content

Treatment with MeJA resulted in a significant increase in the total phenolics of radish microgreens in all the tested cultivars (Figure 2F). Compared to the control group, 1.0 mM MeJA application led to higher rise in total phenolics, except in ‘Asia green 2’ where 0.5 mM MeJA treatment resulted in higher total phenolics than 1.0 mM MeJA. In the control, the total phenolics ranged from 132.80 mg g^−1^ DW in ‘Asia green 2’ to 298.57 mg g^−1^ DW in ‘Koregon red’. However, with the application of MeJA total phenolics ranged from 152.14 mg g^−1^ DW in ‘Asia green 2’ to 335.98 mg g^−1^ DW in ‘Koregon red’, and from 141.16 mg g^−1^ DW in ‘Asia green 2’ to 339.46 mg g^−1^ DW in ‘Koregon red’ at 0.5 and 1.0 mM concentrations, respectively. In addition, ‘Koregon red’ and ‘Asia purple’ appeared to have higher total phenolics than the other three cultivars in both the control and MeJA-treated groups (Figure 2F). Similarly, the application of MeJA consistently increased the content of total flavonoids in all tested microgreen cultivars compared to the control, and treatment with 1.0 mM concentration resulted in highest total flavonoid content in all five cultivars. In the control group, the total flavonoids ranged from 18.14 mg g^−1^ DW in ‘Asia green 1’ to 31.96 mg g^−1^ DW in ‘Koregon red’. However, with the application of MeJA total flavonoids ranged from 29.37 mg g^−1^ DW in ‘Asia green 1’ to 44.67 mg g^−1^ DW in ‘Koregon red’, and from 96.13 mg g^−1^ DW in ‘Asia green 1’ to 109.23 mg g^−1^ DW in ‘Asia purple’ at 0.5 mM and 1.0 mM concentrations, respectively (Figure 2G). The present findings align with those of Mlinarić et al. [45], who observed differently colored radish microgreens cultivated on a substrate and quartz sand mixture under varying light conditions. Additionally, our results regarding the total phenolics and flavonoids from microgreens of the five radish cultivars grown without medium are consistent with the findings reported by Yadav et al. [46]. In their study comparing the microgreens of nine summer season vegetables, they reported total phenolic and flavonoid contents of 1.36 and 3.98 mg g^−1^ FW, respectively, for radish microgreens of the ‘Aoush’ cultivar cultivated on coco pith, perlite, and vermiculite mixture.

#### 3.3.4. Vitamin C Content

The application of MeJA treatment had varying effects on vitamin C content, which were specific to each cultivar, as illustrated in Figure 2H. Among the cultivars studied, ‘Asia purple’ displayed a linear increase in vitamin C content from 201.90 mg 100 g^−1^ to 289.36 mg 100 g^−1^ DW as the concentration of MeJA increased from 0.5 mM to 1.0 mM. Conversely, for ‘Asia green 2’ vitamin C content displayed an inverse pattern, decreasing from 188.90 mg 100 g^−1^ to 133.98 mg 100 g^−1^ DW as the concentration of MeJA increased from 0.5 mM to 1.0 mM. For the remaining three cultivars, the MeJA treatment at both 0.5 mM and 1.0 mM concentrations resulted in a reduction of vitamin C content compared to the control groups (Figure 2H). These distinct responses suggest that the impact of MeJA on vitamin C accumulation is highly dependent on the individual characteristics and genetic makeup of each cultivar. In a study conducted by Yadav et al. [46], microgreens of nine summer season vegetables were compared, revealing that the highest content of ascorbic acid (52.31 mg 100 g^−1^ FW) was observed in ‘Aoush’ radish microgreens cultivated using coco pith, perlite, and vermiculite mixture. Similarly, Mlinarić et al. [45] found variations in vitamin C content ranging from 3.0 to 7.5 mg 100 g^−1^ FW when examining red, green, and purple radish microgreens grown on a substrate and quartz sand mixture under different light conditions. The contents of vitamin C from the microgreens of the tested five cultivars grown without medium exceed the levels reported by Mlinarić et al. [45] and Yadav et al. [46], implying cultivar-specific variation of vitamin C in radish microgreens.

### 3.4. Antioxidant Capacity

The antioxidant capacity of the preharvest MeJA-treated and control microgreens of five radish cultivars that were measured by DPPH, ABTS, and FRAP assays are shown in Table 4. The results revealed that the preharvest MeJA treatment resulted in a significant increase in the antioxidant capacity of radish microgreens across all cultivars. Similar findings were reported by Wang et al. [9] in a study of two cultivars of broccoli florets, where MeJA treatment also showed a remarkable increase in antioxidant capacity. Among the radish microgreens, ‘Asia purple’ treated with 1.0 mM MeJA exhibited the highest antioxidant activity, and ‘Koregon red’ showed the second highest in all assays when the sample concentrations were 1 and 2.5 mg mL^−1^ (Table 4). However, when the sample concentration was increased to 5 mg mL^−1^, the DPPH assay indicated that both ‘Asia purple’ and ‘Koregon red’ had significantly higher antioxidant capacity with 0.5 and 1.0 mM MeJA treatment (Table 4). The range of the DPPH antioxidant capacity of the radish microgreens varied from 83.6 μmol TEAC in control ‘Asia green 2’ to 165.0 and 156.8 μmol TEAC in 1.0 mM MeJA-treated ‘Asia purple’ and ‘Koregon red’, respectively, at 1mg mL^−1^ (Table 4). Similarly, the DPPH antioxidant capacity ranged from 147.2 μmol TEAC in control ‘Asia green 2’ to 301.3 and 301.2 μmol TEAC in 1.0 mM MeJA-treated ‘Asia purple’ and ‘Koregon red’, respectively, at 5 mg mL^−1^ (Table 4). On the other hand, at 5 mg mL^−1^ sample concentration, the ABTS and FRAP assays showed the highest antioxidant capacity in ‘Koregon red’ with 1.0 mM MeJA treatment and in ‘Asia purple’ with 0.5 mM MeJA treatment (Table 4). In all three assays, the values showed a simultaneous increase with the rise in sample concentration. Similarly, ‘Asia purple’ and ‘Koregon red’ displayed significantly higher values in both ABTS and FRAP (Table 4). Overall, the trends demonstrated that MeJA treatment at both 0.5 mM and 1.0 mM concentrations significantly enhanced the antioxidant capacity of all radish microgreen cultivars. MeJA is a compound that can activate plant defense mechanisms in response to several biotic and abiotic stresses [47,48]. It functions as a potential airborne signaling molecule, facilitating intra- and inter-plant communications and triggering defense responses, including antioxidant systems [11,49]. The choice of specific cultivars, such as ‘Asia purple’ and ‘Koregon red’, could further amplify the benefits of the treatment. This study highlights the potential of preharvest MeJA treatment as a strategy to boost the antioxidant properties of radish microgreens, and it offers insights into selecting cultivars with superior antioxidant responses for maximum advantage. As indicated in Section 3.3, preharvest MeJA treatment enhanced total GSLs, total phenolics, total flavonoids and anthocyanins in both ‘Koregon red’ and ‘Asia purple’, and vitamin C in ‘Asia purple’ (Figure 2 and Table 3). Hence, the higher antioxidant capacity of the two cultivars implies the most protective effect of these secondary metabolites with their cumulative or synergetic effects [50]. Generally, as antioxidant capacity primarily reveals the nutritional quality of microgreens, MeJA-treated ‘Asia purple’ and ‘Koregon red’ could be grown as value-added superior cultivars to grow microgreens on trays without media.

### 3.5. Principal Component and Correlation Analysis

For the measured parameters, principal component analysis (PCA) was carried out to acquire a thorough understanding of the interrelationships among the cultivars, treatments, and their nutritional quality. The analysis revealed that 50.54% of the overall variance was explained by the first two principal components, with the first principal component elucidating 34.67% of the total variance (Figure S3). The PCA plot and heat map clearly shows that the five tested radish cultivars and treatments were separated from each other based on the collected parameters (Figures S3 and S4). For instance, ‘Asia purple’ and ‘Koregon red’ were contained in the upper and lower portions of the right quadrants for the MeJA-treated and the control groups, respectively, indicating the benefit of MeJA treatment to enhance the essential and total amino acids, total anthocyanins, flavonoids, phenolics, and antioxidant capacity of the two cultivars (Figure S3). On the other hand, the control groups of ‘Asia green 1’, ‘Asia red’, and ‘Asia green 2’ were located in the lower left quadrant of the PCA plot. In contrast, the treated groups were located nearby or in the upper left quadrant, exhibiting lower values compared to the other tested cultivars for most of the measured parameters; however, they showed higher levels of Chls, GSLs, and minerals such as K, Cu, Mo, Zn, Mg, and Mn (Figure S3). In addition, the correlation coefficients between antioxidant capacity and total phenolics were 0.91, 0.88, and 0.97 DPPH, ABTS, and FRAP assays, respectively. Anthocyanins also exhibited stronger positive correlation coefficients of 0.87, 0.83, and 0.96 with DPPH, ABTS, and FRAP assays, respectively (Figure S5). These findings indicate that total anthocyanins and phenolics are the primary contributors of antioxidant capacity in radish microgreens (Figures S3–S5).

## 4. Conclusions

This study examined how preharvest MeJA treatment affected the nutritional quality and yield of five radish cultivars grown as microgreens using a substrate-free sprouting technique in a vertical growing unit. Microgreens were treated with a foliar application of 0.5 mM and 1.0 mM MeJA on the 7th day, three days before harvest. The study evaluated parameters such as fresh weight, dry matter percentage, mineral content, amino acids, secondary metabolites (including chlorophylls, anthocyanins, vitamin C, phenolics, flavonoids, and GSLs), and antioxidant capacity.

The findings indicated that 1.0 mM MeJA generally improved yield and dry weight across cultivars, and all microgreens demonstrated substantial mineral and amino acid compositions, with the influence of cultivar being more significant than MeJA treatment. However, MeJA treatment notably enhanced anthocyanins, GSLs, phenolics, flavonoids, and antioxidant capacity of all cultivars.

Generally, the results of this study suggest that MeJA treatment has the potential to increase secondary metabolites in radish microgreens. Moreover, it was evident that distinct cultivars exhibited varying responses to MeJA treatment among the five radish cultivars. Additionally, the cultivation method employed in this study to produce microgreens without substrate could be a viable strategy for screening cultivars and meeting the nutritional and functional requirements without environmental impacts.

The findings underscore the benefits of supplementing MeJA treatment with a substrate-free microgreens production method to enhance the quality of radish microgreens. The improvement in quality arises from the stimulation of secondary metabolites, which leads to increased antioxidant capacity, ultimately yielding value-added microgreens. Consequently, MeJA-treated ‘Asia purple’ and ‘Koregon red’ cultivars emerge as promising candidates to produce value-added microgreens on trays without substrate. Future research could explore the effects of prolonged and repeated MeJA applications on the nutritional quality of microgreens grown from different seed varieties.

## Figures and Tables

**Table 1:** Mineral contents of five radish microgreen cultivars, cultivated without substrate and harvested on the 10th day, influenced by the interaction between the cultivars and preharvest MeJA treatment at 0.5 mM and 1.0 mM concentrations applied on the 7th day post-sowing.

Minerals (mg kg^−1^) DW	Asia Green 1			Asia Green 2			Asia Red			Koregon Red			Asia Purple			Significance Level		
	Control	0.5 mM MeJA	1.0 mM MeJA	Control	0.5 mM MeJA	1.0 mM MeJA	Control	0.5 mM MeJA	1.0 mM MeJA	Control	0.5 mM MeJA	1.0 mM MeJA	Control	0.5 mM MeJA	1.0 mM MeJA	Cultivr (A)	Treatment (B)	A × B
N	55,770 ± 40 e	55,170 ± 53 h	55,080 ± 97 i	47,410 ± 83 n	50,775 ± 80 l	48,675 ± 93 m	46,840 ± 78 o	56,490 ± 62 d	58,685 ± 43 b	65,685 ± 63 a	57,690 ± 63 c	55,355 ± 39 f	53,875 ± 65 k	54,285 ± 49 j	55,295 ± 43 g	***	***	***
P	9459 ± 8 o	9722 ± 67 n	9781 ± 48 m	10,575 ± 67 j	11,519 ± 7 c	11,787 ± 43 a	10,251 ± 72 k	10,877 ± 22 g	10,183 ± 5 l	10,935 ± 5 f	11,594 ± 28 b	11,164 ± 66 e	10,806 ± 13 h	11,174 ± 7 d	10,740 ± 61 i	***	***	***
K	7890 ± 4 i	7148 ± 68 m	6883 ± 31 n	9568 ± 55 d	7231 ± 3 k	8120 ± 35 h	10,372 ± 59 a	9906 ± 53 b	9706 ± 4 c	7221 ± 4 l	8404 ± 51 g	7353 ± 62 j	8526 ± 65 f	8720 ± 5 e	6650 ± 59 o	***	***	***
Ca	4040 ± 12 h	4244 ± 41 g	3997 ± 10 i	3547 ± 63 n	3552 ± 21 m	3658 ± 12 k	3536 ± 64 o	3648 ± 38 l	3692 ± 2 j	4698 ± 3 f	5282 ± 87 b	5141 ± 12 c	5034 ± 42 d	5395 ± 13 a	4822 ± 63 e	***	***	***
Mg	3474 ± 21 k	3615 ± 12 d	3600 ± 29 e	3548 ± 23 h	3873 ± 16 a	3872.34 a	3511 ± 25 j	3817 ± 38 b	3532.91 i	3404 ± 20 m	3577 ± 36 f	3629.89 c	3433 ± 14 l	3558 ± 32 g	3509.50 j	***	***	***
Na	1414 ± 3.2 b	1412 ± 2.9 b	1083 ± 3.4 i	1208 ± 7.1 e	915 ± 2.2 m	9880 ± 5.6 k	1121 ± 6.8 h	956 ± 1.8 l	1015 ± 2.4 j	1179 ± 3.7 g	1304 ± 1.9 d	1460 ± 6.9 a	1194 ± 3.1 f	1313 ± 1.3 c	1196 ± 7.3 f	***	***	***
Mn	23.6 ± 0.1 l	24.4 ± 4.1 k	24.9 ± 0.1 j	30.9 ± 2.4 c	35.6 ± 0.1 a	35.0 ± 0.1 b	18.6 ± 2.5 o	21.4 ± 2.3 m	20.7 ± 0.1 n	25.6 ± 0.1 h	27.1 ± 2.3 d	26.5 ± 0.3 f	25.3 ± 4.1 i	26.1 ± 0.1 g	26.7 ± 0.3 e	***	***	***
Fe	67.67 ± 0.3 k	73.59 ± 1.0 f	71.27 ± 3.1 g	59.99 ± 0.3 m	69.53 ± 0.1 g	64.21 ± 3.1 l	57.00 ± 0.3 o	68.13 ± 0.7 j	58.96 ± 0.1 n	77.83 ± 0.1 b	77.16 ± 0.7 c	74.49 ± 1.6 e	68.52 ± 1.03 i	74.98 ± 0.1 d	79.16 ± 1.7 a	***	***	***
Cu	3.37 ± 0.1 b	3.40 ± 0.3 b	3.38 ± 0.34 b	2.35 ± 0.3 f	2.27 ± 0.1 g	2.33 ± 0.3 f	3.25 ± 0.3 d	3.33 ± 0.1 c	3.85 ± 0.1 a	2.48 ± 0.1 e	1.70 ± 0.3 i	1.54 ± 0.6 j	1.56 ± 0.1 j	1.35 ± 0.1 k	1.78 ± 0.6 h	***	***	***
Zn	31.12 ± 0.1 i	41.30 ± 0.1 a	32.41 ± 0.3 g	27.89 ± 1.1 m	36.29 ± 0.1 b	34.09 ± 0.1 d	27.32 ± 1.1 o	35.42 ± 0.1 c	33.71 ± 0.1 e	27.65 ± 0.1 n	29.66 ± 0.1 j	32.90 ± 0.4 f	28.55 ± 0.23 l	31.35 ± 0.1 h	28.86 ± 0.4 k	***	***	***
Mo	1.51 ± 0.1 a	1.44 ± 0.2 b	1.41 ± 0.1 c	0.26 ± 0.4 g	0.26 ± 0.1 g	0.27 ± 0.1 g	0.78 ± 0.4 e	0.81 ± 0.2 d	0.74 ± 0.1 f	0.08 ± 0.1 i	0.07 ± 0.2 i	0.02 ± 0.4 j	0.12 ± 0.2 h	0.02 ± 0.1 j	0.02 ± 0.4 j	***	***	***
Total	82,174 ± 59 i	81,455 ± 125 j	80,558 ± 347 k	75,979 ± 212 n	78,009 ± 96 l	77,238 ± 342 m	75,739 ± 207 o	85,825 ± 577 d	86,932 ± 59 c	93,256 ± 76 a	87,988 ± 584 b	84,239 ± 56 f	82,994 ± 16 g	84,581 ± 66 e	82,351 ± 52 h	***	***	***

The reported values shown are three replicates’ mean ± standard errors. Significant differences at *p* < 0.001 are indicated by ***. Different letters within the same row indicate significant interaction differences between cultivars and treatments with Duncan’s mean separation at α = 0.05.

**Table 2:** The amino acid content of five radish microgreen cultivars, cultivated without substrate and harvested on the 10th day, influenced by the interaction between the cultivars and preharvest MeJA treatment at 0.5 mM and 1.0 mM concentration applied on the 7th day post-sowing.

Amino Acids (mg kg^−1^) DW	Asia Green 1			Asia Green 2			Asia Red			Koregon Red			Asia Purple		
	Control	0.5 mM MeJA	1.0 mM MeJA	Control	0.5 mM MeJA	1.0 mM MeJA	Control	0.5 mM MeJA	1.0 mM MeJA	Control	0.5 mM MeJA	1.0 mM MeJA	Control	0.5 mM MeJA	1.0 mM MeJA
Aspartic acid	605 ± 7.4 g	668 ± 4.7 e	680 ± 8.9 e	567 ± 10 i	590 ± 4.1 gh	575 ± 2.6 hi	665 ± 1.7 e	707 ± 3.1 d	680 ± 2.3 e	635 ± 0.6 f	843 ± 4.6 a	747.23 ± 6.9 c	724 ± 4.4 d	846 ± 0.6 a	776 ± 13 b
Glutamic acid	2545 ± 12 de	2443 ± 25 gh	2628 ± 25 c	2114 ± 18 i	2016 ± 13 j	2017 ± 4.6 j	2523 ± 5.9 ef	2426 ± 11 h	2486 ± 16 fg	2580 ± 8.6 cd	2619 ± 15 c	2683 ± 15 b	2705 ± 8.2 ab	2736 ± 3.2 a	2456 ± 36 gh
Asparagine	4479 ± 11 e	4465 ± 31 e	3681 ± 9.3 h	2862 ± 44 j	2380 ± 14 k	2453 ± 1.1 k	5012 ± 16 c	4736 ± 21 d	4668 ± 21 d	5530 ± 20 a	4443 ± 13 e	5116 ± 15 b	4007 ± 11 f	3785 ± 10 g	2950 ± 35 i
Serine	1400 ± 2.8 d	1343 ± 10 e	1547 ± 2.9 c	1313 ± 19 f	1060 ± 7.9 hi	1061 ± 1.1 hi	1125 ± 1.3 g	1004 ± 3.6 j	899 ± 3.9 k	1079 ± 11 h	864 ± 7.7 l	1041 ± 3.5 i	1731 ± 1.7 a	1669 ± 1.6 b	1544 ± 8.9 c
Glutamine	34,484 ± 11 b	28,867 ± 23 g	30,138 ± 69 f	29,830 ± 31 f	22,449 ± 14 l	24,024 ± 78 k	33,311 ± 4.1 c	27,077 ± 89 h	26,712 ± 91 h	37,506 ± 19 a	25,241 ± 94 j	31,029 ± 79 e	32,835 ± 19 d	26,077 ± 44 i	21,472 ± 66 m
Histidine (E)	5108 ± 12 ef	5273 ± 46 bc	5141 ± 6.4 de	4942 ± 5.8 fg	5059 ± 62 ef	4798 ± 28 g	5175 ± 39 cd	5326 ± 39 ab	4535 ± 23 h	5483 ± 36 a	5279 ± 44 bc	4773 ± 18 g	4956 ± 39 fg	5478 ± 37 a	4945 ± 19 fg
Glycine	450 ± 1.6 a	390 ± 1.2 c	413 ± 17 b	286 ± 8.1 d	285 ± 5.9 d	259 ± 5.8 e	266 ± 8.5 de	201 ± 1.6 f	179 ± 0.9 g	281 ± 8.1 d	188 ± 6.1 fg	205 ± 3.6 f	467 ± 3.7 a	458 ± 1.7 a	393 ± 0.5 c
Threonine (E)	2241 ± 1.8 d	2133 ± 8.2 e	2287 ± 28 d	2011 ± 27 f	1872 ± 14 h	1897 ± 5.5 h	1960 ± 25 g	1545 ± 6.2 k	1598 ± 26 j	2094 ± 13 e	1354 ± 9.4 l	1782 ± 5.6 i	2820 ± 11 a	2570 ± 5.3 b	2389 ± 3.3 c
Citrulline	80 ± 0.3 d	58 ± 1.3 g	72 ± 3.2 e	57 ± 0.53 g	45 ± 2.8 h	87 ± 0.4 c	72 ± 1.5 e	66 ± 2.5 f	82 ± 1.1 cd	81 ± 0.7 d	74 ± 3.1 e	93 ± 1.9 b	110 ± 2.2 a	85 ± 2.6 cd	108 ± 0.6 a
Arginine	1203 ± 6.6 h	851 ± 11 i	704 ± 4.6 k	786 ± 9.1 j	544 ± 4.9 l	484 ± 6.7 m	3356 ± 4.6 b	2686 ± 12 d	2219 ± 19 g	2960 ± 27 c	2262 ± 21 f	2668 ± 21 d	3849 ± 9.2 a	2950 ± 4.1 c	2371 ± 13 e
Alanine	1196 ± 4.7 b	864 ± 5.2 h	1036 ± 11 e	1152 ± 13 c	699 ± 5.1 k	824 ± 2.2 i	1191 ± 0.5 b	823 ± 2.5 i	695 ± 4.3 k	1091 ± 6.1 d	773 ± 5.6 j	769 ± 2.7 j	1405 ± 2.9 a	984 ± 0.9 f	907 ± 7.1 g
GABA	159 ± 0.9 e	149 ± 0.5 fg	138 ± 3.1 h	172 ± 3.3 d	138 ± 1.3 h	159 ± 2.9 e	148 ± 2.4 fg	153 ± 0.5 ef	108 ± 1.1 i	173 ± 4.1 d	144 ± 1.4 gh	110 ± 1.7 i	218 ± 0.6 c	262 ± 1.4 a	244 ± 2.9 b
Tyrosine	302 ± 1.1 j	257 ± 2.8 l	276 ± 2.9 k	368 ± 3.4 i	281 ± 2.5 k	278 ± 0.3 k	671 ± 0.4 cd	449 ± 1.8 f	393 ± 3.5 g	678 ± 5.4 c	377 ± 6.1 h	514 ± 1.3 e	884 ± 1.7 a	748 ± 0.7 b	668 ± 3.1 d
Valine (E)	1425 ± 3.3 h	1406 ± 13 h	1611 ± 21 f	1297 ± 9.8 j	1029 ± 6.1 m	1095 ± 1.2 l	1844 ± 4.9 d	1346 ± 4.1 i	1253 ± 6.8 k	1792 ± 8.6 e	1097 ± 4.6 l	1534 ± 11 g	2392 ± 6.2 a	2111 ± 1.4 b	2039 ± 13 c
Methionine (E)	120 ± 1.4 b	100 ± 1.4 e	111 ± 3.1 cd	77 ± 1.1 h	73 ± 0.8 hi	70 ± 0.1 i	93 ± 1.3 f	77 ± 0.1 h	75 ± 0.2 h	108 ± 1.1 d	87 ± 0.6 g	76 ± 0.3 h	135 ± 1.1 a	120 ± 0.7 b	113 ± 2.2 c
Tryptophane (E)	358 ± 2.2 i	358 ± 3.3 i	281 ± 8.5 k	303 ± 3.3 j	270 ± 1.2 k	249 ± 3.2 l	621 ± 7.3 b	416 ± 2.7 f	392 ± 1.6 gh	748 ± 1.1 a	384 ± 1.4 h	445 ± 5.9 e	575 ± 3.2 c	494 ± 0.9 d	402 ± 7.6 g
Phenylalanine (E)	707 ± 2.4 e	844 ± 11 d	626 ± 5.1 f	527 ± 2.2 h	573 ± 5.5 g	584 ± 0.9 g	469 ± 2.4 i	385 ± 1.9 k	372 ± 2.9 k	530 ± 8.7 h	427 ± 7.8 j	385 ± 9.3 k	1154 ± 0.8 b	1230 ± 1.6 a	931 ± 3.1 c
Isoleucine (E)	2673 ± 10 j	2834 ± 34 i	3175 ± 45 h	2837 ± 48 i	3438 ± 31 g	2913 ± 40 i	4470 ± 44 f	5209 ± 27 c	5527 ± 54 b	4690 ± 76 e	5150 ± 44 c	5714 ± 31 a	4481 ± 17 f	5697 ± 22 a	4868 ± 19 d
Ornitnine	321 ± 1.4 cd	337 ± 4.5 bc	351 ± 19 b	310 ± 4.4 d	340 ± 1.2 b	273 ± 4.5 e	310 ± 6.5 d	312 ± 1.2 d	274 ± 4.1 e	302 ± 2.8 d	389 ± 2.5 a	314 ± 4.6 d	273 ± 5.3 e	350 ± 0.6 b	303 ± 1.8 d
Leucine (E)	154 ± 1.8 f	132 ± 1.9 h	143 ± 1.5 g	152 ± 1.4 f	100 ± 0.2 j	89 ± 0.3 k	239 ± 0.8 b	152 ± 3.1 f	145 ± 1.3 g	238 ± 1.3 b	118 ± 0.6 i	196 ± 1.3 e	283 ± 1.4 a	206 ± 0.2 c	201 ± 1.2 d
Lysine (E)	332 ± 3.6 gh	254 ± 8.6 ij	253 ± 14 ij	356 ± 8.3 gh	306 ± 7.1 hi	229 ± 12 j	930 ± 17 a	637 ± 9.8 c	426 ± 16 ef	750 ± 15 b	471 ± 8.8 d	461 ± 18 de	674 ± 13 c	486 ± 4.2 d	389 ± 5.5 fg
Proline	269 ± 9.6 cde	242 ± 0.7 e	250 ± 9.9 de	225 ± 6.9 ef	177 ± 6.7 f	237 ± 8.7 e	341 ± 6.4 ab	184 ± 8.1 f	267 ± 2.9 cde	322 ± 6.8 ab	366 ± 6.9 a	222 ± 2.1 ef	298 ± 0.7 bcd	250 ± 0.1 de	307 ± 5.8 bc
Total E	13,122 ± 11 h	13,339 ± 56 gh	13,631 ± 58 g	12,506 ± 40 i	12,724 ± 47 i	11,928 ± 18 j	15,804 ± 12 d	15,098 ± 66 e	14,327 ± 29 f	16,438 ± 60 c	14,372 ± 78 f	15,370 ± 19 e	17,476 ± 36 b	18,395 ± 64 a	16,279 ± 83 c
Total amino acids	60,622 ± 84 d	54,280 ± 79 g	55,553 ± 58 f	52,554 ± 78 h	43,734 ± 31 k	44,667 ± 10 j	64,802 ± 5 c	55,927 ± 25 f	53,994 ± 35 g	69,663 ± 61 a	52,964 ± 55 h	60,886 ± 68 d	66,988 ± 74 b	59,600 ± 68 e	50,785 ± 56 i
Cultivar (A)	***	***	***	***	***	***	***	***	***	***	***	***	***	***	***
Treatment (B)	***	***	***	***	***	***	***	***	***	***	***	***	***	***	***
AXB	***	***	***	***	***	***	***	***	***	***	***	***	***	***	***

E represents essential amino acid. Amino acid content is presented as the mean of three replicates. *** and AXB indicate significant differences at *p* < 0.001 and interaction between cultivars and treatments. Different letters within the same row indicate significant interaction differences between cultivars and treatments with Duncan’s mean separation at α = 0.05.

**Table 3:** The individual and total glucosinolates content of five radish microgreen cultivars, cultivated without substrate and harvested on the 10th day, influenced by the interaction between the cultivars and preharvest MeJA treatment at 0.5 mM and 1.0 mM concentration applied on the 7th day post-sowing.

Glucosinolates (mg g^−1^ DW)	Asia Green 1			Asia Green 2			Asia Red			Koregon Red			Asia Purple		
	Control	0.5 mM MeJA	1.0 mM MeJA	Control	0.5 mM MeJA	1.0 mM MeJA	Control	0.5 mM MeJA	1.0 mM MeJA	Control	0.5 mM MeJA	1.0 mM MeJA	Control	0.5 mM MeJA	1.0 mM MeJA
Glucoiberin	0.26 ± 0.04 c	0.04 ± 0.01 c	23.35 ± 0.48 a	0.32 ± 0.06 c	0.04 ± 0.01 c	23.44 ± 0.44 a	0.22 ± 0.10 c	0.04 ± 0.01 c	13.18 ± 0.61 b	0.25 ± 0.11 c	0.03 ± 0.01 c	20.37 ± 0.33 a	0.27 ± 0.03 c	0.04 ± 0.01 c	21.93 ± 0.15 a
Glucoraphenin	3.87 ± 0.16 de	4.64 ± 0.25 d	1.03 ± 0.01 g	1.86 ± 0.13 fg	3.81 ± 0.23 de	1.09 ± 0.01 fg	1.37 ± 0.19 fg	3.93 ± 0.15 de	2.69 ± 0.16 ef	14.05 ± 0.65 ab	11.78 ± 0.63 c	2.56 ± 0.10 fg	12.72 ± 0.82 bc	14.64 ± 0.54 a	2.42 ± 0.12 fg
Gluconapin	0.01 ± 0.00 ab	0.02 ± 0.01 a	0.01 ± 0.00 ab	0.00 ± 0.00 b	0.01 ± 0.0 ab	0.00 ± 0.0 b	0.00 ± 0.0 b	0.00 ± 0.0 b	0.01 ± 0.0 ab	0.00 ± 0.0 b	0.00 ± 0.0 b	0.01 ± 0.0 ab	0.00 ± 0.0 b	0.00 ± 0.0 b	0.01 ± 0.01 ab
4-Hydroxyglucobrassicin	0.60 ± 0.03 c	1.12 ± 0.04 ab	0.13 ± 0.01 d	0.65 ± 0.03 c	1.25 ± 0.04 a	0.13 ± 0.02 d	0.64 ± 0.02 c	1.36 ± 0.05 a	0.73 ± 0.03 bc	0.78 ± 0.03 bc	0.99 ± 0.05 abc	0.13 ± 0.01 d	0.93 ± 0.03 abc	0.81 ± 0.01 bc	0.16 ± 0.03 d
Glucoerucin	0.67 ± 0.02 de	1.17 ± 0.05 b	1.64 ± 0.02 a	0.67 ± 0.12 d	1.18 ± 0.05 b	1.15 ± 0.05 bc	1.08 ± 0.02 bc	1.08 ± 0.03 bc	1.16 ± 0.04 b	0.48 ± 0.02 e	0.68 ± 0.00 d	1.07 ± 0.01 bc	0.53 ± 0.02 de	0.63 ± 0.00 de	0.97 ± 0.01 c
Glucoraphastin	30.77 ± 0.26 bc	31.92 ± 0.68 bc	60.19 ± 1.42 a	27.70 ± 0.45 c	31.42 ± 0.55 bc	34.50 ± 1.76 b	34.74 ± 0.73 b	28.31 ± 0.61 c	34.70 ± 1.59 b	16.89 ± 0.08 d	20.11 ± 0.48 d	31.08 ± 0.80 bc	17.92 ± 0.67 d	17.46 ± 0.21 d	28.75 ± 1.41 c
Glucobrassicin	0.34 ± 0.12 g	1.13 ± 0.08 ef	1.01 ± 0.01 ef	0.60 ± 0.05 fg	2.42 ± 0.11 b	1.75 ± 0.22 cd	0.29 ± 0.01 g	1.72 ± 0.11 cd	1.53 ± 0.21 de	0.32 ± 0.03 g	1.99 ± 0.05 cd	3.40 ± 0.29 a	0.38 ± 0.03 g	1.90 ± 0.04 cd	2.06 ± 0.07 bc
4-Methoxyglucobrassicin	2.06 ± 0.05 b	2.65 ± 0.14 a	0.87 ± 0.01 g	1.74 ± 0.13 def	2.81 ± 0.11 a	0.81 ± 0.08 g	1.45 ± 0.04 f	2.02 ± 0.11 bc	0.87 ± 0.33 g	1.58 ± 0.01 def	1.89 ± 0.06 bcd	0.71 ± 0.04 g	1.50 ± 0.06 ef	1.81 ± 0.02 cde	0.72 ± 0.03 g
TGSLs	38.57 ± 0.24 cde	42.69 ± 0.36 cd	88.22 ± 1.86 a	33.54 ± 0.59 e	42.94 ± 0.64 c	62.87 ± 2.65 b	39.80 ± 0.60 cde	38.47 ± 0.47 cde	54.86 ± 3.63 b	34.35 ± 0.74 de	37.47 ± 1.10 cde	59.33 ± 2.84 b	34.26 ± 1.54 de	37.30 ± 0.49 cde	57.01 ± 4.12 b
Cultivar (A)	***	***	***	***	***	***	***	***	***	***	***	***	***	***	***
Treatment (B)	***	***	***	***	***	***	***	***	***	***	***	***	***	***	***
AXB	***	***	***	***	***	***	***	***	***	***	***	***	***	***	***

Different letters within the same row indicate significant interaction differences between cultivars and treatments with Duncan’s mean separation at α = 0.05. *** and AXB indicate significant differences at *p* < 0.001 and the interaction between cultivars and treatments.

**Table 4:** The antioxidant capacity with 2, 2-di-phenyl-1-picrylhydrazyl radical scavenging capacity (DPPH), trolox-equivalent antioxidant capacity (ABTS), and ferric-reducing antioxidant power (FRAP) at 1.0, 2.5, and 5 mg L^−1^ from five radish microgreen cultivars, cultivated without substrate and harvested on the 10th day, influenced by the interaction between the cultivars and preharvest MeJA treatment at 0.5 mM and 1.0 mM concentration applied on the 7th day post-sowing.

Parameters/Sample Concentration	Treatments	Cultivars				
		Asia Green 1	Asia Green 2	Asia Red	Koregon Red	Asia Purple
DPPH 1.0 mg mL^−1^	Control	107.9 ± 3.8 def	83.6 ± 5.7 g	95.9 ± 6.2 fg	139.8 ± 3.1 c	148.0 ± 2.3 bc
	0.5 MeJA	109.5 ± 1.4 de	96.5 ± 3.0 f	98.0 ± 1.6 ef	149.8 ± 2.7 bc	155.2 ± 9.4 ab
	1 MeJA	111.9 ± 0.6 d	99.5 ± 4.9 ef	105.4 ± 3.7 def	156.8 ± 3.3 ab	165.0 ± 2.8 a
DPPH 2.5 mg mL^−1^	Control	142.5 ± 4.3 ef	107.2 ± 3.8 h	124.1 ± 1.1 g	204.4 ± 0.9 c	210.0 ± 2.0 c
	0.5 MeJA	151.9 ± 3.0 d	125.6 ± 3.3 g	135.1 ± 1.7 f	226.1 ± 0.3 b	238.7 ± 0.3 a
	1 MeJA	155.1 ± 3.6 d	127.0 ± 4.1 g	143.3 ± 3.8 b	224.4 ± 2.0 b	244.4 ± 1.4 a
DPPH 5 mg mL^−1^	Control	196.2 ± 2.0 e	147.6 ± 3.0 h	178.8 ± 0.2 f	286.1 ± 0.1 b	288.8 ± 0.1 b
	0.5 MeJA	209.9 ± 2.3 d	171.0 ± 0.6 g	195.6 ± 2.4 e	298.4 ± 0.6 a	301.1 ± 0.3 a
	1 MeJA	221.8 ± 1.5 c	177.3 ± 2.4 f	198.2 ± 3.3 e	301.2 ± 0.2 ba	301.3 ± 0.7 a
ABTS 1 mg mL^−1^	Control	6.0 ± 0.2 ef	5.3 ± 0.1 g	5.2 ± 0.2 g	7.8 ± 0.2 c	7.7 ± 0.3 c
	0.5 MeJA	7.0 ± 0.2 d	5.6 ± 0.1 fg	6.4 ± 0.2 e	8.9 ± 0.2 b	9.4 ± 0.3 ab
	1 MeJA	7.7 ± 0.2 c	6.0 ± 0.1 ef	6.5 ± 0.3 de	9.4 ± 0.2 ab	9.6 ± 0.1 a
ABTS 2.5 mg mL^−1^	Control	9.2 ± 0.1 gh	6.8 ± 0.3 j	7.7 ± 0.3 i	14.1 ± 0.4 c	13.3 ± 0.2 d
	0.5 MeJA	10.0 ± 0.2 f	9.5 ± 0.1 fg	8.0 ± 0.1 i	15.2 ± 0.1 b	15.5 ± 0.1 b
	1 MeJA	11.1 ± 0.1 e	7.7 ± 0.3 i	8.9 ± 0.2 h	16.3 ± 0.1 a	15.8 ± 0.2 ab
ABTS 5 mg mL^−1^	Control	13.9 ± 0.1 h	10.8 ± 0.3 i	14.3 ± 0.2 gh	22.4 ± 0.2 d	22.7 ± 0.3 d
	0.5 MeJA	16.2 ± 0.1 f	16.3 ± 0.2 f	14.8 ± 0.1 g	26.1 ± 0.2 c	27.2 ± 0.1 b
	1 MeJA	17.3 ± 0.2 e	13.9 ± 0.3 h	14.2 ± 0.1 gh	28.0 ± 0.3 a	26.1 ± 0.1 c
FRAP 1 mg mL^−1^	Control	13.6 ± 0.1 j	11.4 ± 0.1 k	14.2 ± 0.1 ij	25.9 ± 0.1 e	27.2 ± 0.1 d
	0.5 MeJA	16.1 ± 0.2 fg	13.5 ± 0.1 j	14.7 ± 0.1 hi	28.4 ± 0.2 c	29.3 ± 0.2 bc
	1 MeJA	16.7 ± 0.5 f	13.4 ± 0.2 j	15.4 ± 0.1 gh	29.5 ± 0.3 b	31.6 ± 0.9 a
FRAP 2.5 mg mL^−1^	Control	26.4 ± 0.2 i	22.6 ± 0.1 k	25.1 ± 0.1 j	47.6 ± 0.7 d	49.9 ± 0.2 c
	0.5 MeJA	29.7 ± 0.1 ef	27.5 ± 0.1 h	28.1 ± 0.4 gh	54.5 ± 0.3 b	58.9 ± 0.2 a
	1 MeJA	30.4 ± 0.2 e	25.2 ± 0.2 j	28.7 ± 0.3 fg	58.7 ± 0.6 a	59.4 ± 0.3 a
FRAP 5 mg mL^−1^	Control	51.0 ± 0.5 j	45.0 ± 0.1 k	50.4 ± 0.2 j	93.6 ± 0.8 e	95.8 ± 0.8 d
	0.5 MeJA	56.2 ± 0.4 h	51.4 ± 0.7 j	57.7 ± 0.3 g	102.1 ± 0.1 c	108.8 ± 0.6 a
	1 MeJA	61.6 ± 0.3 f	51.5 ± 0.2 j	54.6 ± 0.3 i	109.6 ± 0.1 a	106.2 ± 0.1 b
Significance (*p*)	Cultivar (A)	***	***	***	***	***
	Treatment (B)	***	***	***	***	***
	AXB	***	***	***	***	***

Means with different letters under the same parameter and sample concentration are significantly different (*p* < 0.05) with Duncan’s multiple range test. Antiradical capacity values are in Trolox equivalents (μmol TE for each sample concentration). *** and AXB indicate significant differences at *p* < 0.001 and the interaction between cultivars and treatments.

## Data Availability

The original contributions presented in the study are included in the article/Supplementary Material; further inquiries can be directed to the corresponding author.

## Supplementary Materials

The following supporting information can be downloaded at: https://www.mdpi.com/article/10.3390/foods13050789/s1, Table S1. Average weight of 1000 seeds and seed germinability of five radish cultivars for microgreens. Table S2. The arrangement of the treatment combinations and abbreviations used. Figure S1. Growing tray (A) and harvesting stage (B) of radish microgreens. Figure S2. γ-aminobutyric acid (GABA) (A), total essential amino acids (B), and total amino acids (C) of five radish microgreen cultivars, cultivated without substrate and harvested on the 10th day, influenced by the interaction between the cultivars and preharvest MeJA treatment at 0.5 mM and 1.0 mM concentrations applied on the 7th day post-sowing. Vertical bars indicate average values of three replicates ± standard error. Different letters on the bars indicate significant differences between cultivars at α = 0.05 with Duncan’s mean separation procedure. Figure S3. Biplot of five radish microgreen cultivars, cultivated without substrate and harvested on the 10th day, influenced by the interaction between the cultivars and preharvest MeJA treatment at 0.5 mM and 1.0 mM concentrations applied on the 7th day post-sowing. The data normalization was done by median combined with autoscaling, and these analyses were performed using MetaboAnalyst 5.0 software (https://www.metaboanalyst.ca/). G1, G2, AR, AP, KR, A, B, C, Cha, Chb, TCh, TAA, TEA, VC, TP, TF, Total GSLs, DPPH, FRAP, and ABTS represent ‘Asia green 1’, ‘Asia green 2’, ‘Asia red’, ‘Asia purple’, ‘Koregon red’, treatment A (control), treatment B (0.5-mM MeJA), treatment C (1.0-mM MeJA), chlorophyll a, chlorophyll b, total chlorophyll, total amino acids, total essential amino acids, vitamin C, total phenolics, total flavonoids, total glucosinolates, α-diphenyl-β-picrylhydrazyl, ferric reducing antioxidant power, and 2,2′-azino-bis (3-ethylbenzothiazoline-6-sulfonic acid), respectively. Figure S4. Heat map of five radish microgreen cultivars, cultivated without substrate and harvested on the 10th day, influenced by the interaction between the cultivars and preharvest MeJA treatment at 0.5 mM and 1.0 mM concentrations applied on the 7th day post-sowing. The data normalization was done by median combined with autoscaling, and the analysis was performed using MetaboAnalyst 5.0 software (https://www.metaboanalyst.ca/). G1, G2, AR, AP, KR, A, B, C, Cha, Chb, TCh, TAA, TEA, VC, TP, TF, Total GSLs, DPPH, FRAP, and ABTS represent ‘Asia green 1’, ‘Asia green 2’, ‘Asia red’, ‘Asia purple’, ‘Koregon red’, treatment A (control), treatment B (0.5-mM MeJA), treatment C (1.0-mM MeJA), chlorophyll a, chlorophyll b, total chlorophyll, total amino acids, total essential amino acids, vitamin C, total phenolics, total flavonoids, total glucosinolates, α-diphenyl-β-picrylhydrazyl, ferric reducing antioxidant power, and 2,2′-azino-bis (3-ethylbenzothiazoline-6-sulfonic acid), respectively. Figure S5. Correlation heat map of nutritional quality parameters in five radish microgreen cultivars, cultivated without substrate and harvested on the 10th day, influenced by the interaction between the cultivars and preharvest MeJA treatment at 0.5 mM and 1.0 mM concentrations applied on the 7th day post-sowing. Cha, Chb, TCh, TAA, TEA, VC, TP, TF, Total GSLs, DPPH, FRAP, and ABTS represent chlorophyll a, chlorophyll b, total chlorophyll, total amino acids, total essential amino acids, vitamin C, total phenolics, total flavonoids, total glucosinolates, α-diphenyl-β-picrylhydrazyl, ferric reducing antioxidant power, and 2,2′-azino-bis (3-ethylbenzothiazoline-6-sulfonic acid), respectively.
