# Supplementary material for: Preharvest Methyl Jasmonate Treatment Affects the Mineral Profile, Metabolites, and Antioxidant Capacity of Radish Microgreens Produced without Substrate
Source: Foods. 2024 Mar 4;13(5):789. doi: 10.3390/foods13050789 (PMC10930673; doi:10.3390/foods13050789)
Supplement: Supplementary file 1 [file foods-13-00789-s001.zip › foods-2863504-supplementary.pdf]

Preharvest methyl jasmonate treatment affects the mineral profile, metabolites, and antioxidant activities of radish microgreens produced without substrate

Shimeles Tilahun[1,2], Min Woo Baek[3,4], Ki-Seok An[4,5], Han Ryul Choi[6], Jong Hwan Lee[3,4], Su Ho Tae[3,4], Do Su Park[1], Jin Sung Hong[7,*], Cheon Soon Jeong[3,4,*]

[1]Agriculture and Life Science Research Institute, Kangwon National University, Chuncheon 24341, the Republic of Korea

[2]Department of Horticulture and Plant Sciences, Jimma University, Jimma 378, Ethiopia

[3]Interdisciplinary Program in Smart Agriculture, Kangwon National University, Chuncheon 24341, the Republic of Korea

[4]Department of Horticulture, Kangwon National University, Chuncheon 24341, the Republic of Korea

[5]Kangwon National University Eco-friendly Agricultural Product Safety Center, Chuncheon 24341, the Republic of Korea

[6]National Institute of Horticultural and Herbal Science, Rural Development Administration, Wanju-gun 55365, the Republic of Korea

[7]Department of Applied Biology, Kangwon National University, Chuncheon, the Republic of Korea

***Correspondence:** jinsunghong@kangwon.ac.kr; jeongcs@kangwon.ac.kr

Table S1. Average weight of 1000 seeds and seed germinability of five radish cultivars for microgreens.

Cultivars	1000 seed weight	Germinability
Asia green 1	10.63±0.07c	98±0.58b
Asia green 2	16.21±0.18b	99±0.58a
Asia red	10.22±0.27d	98±1.00b
Koregon red	17.23±0.07a	98±1.00b
Asia purple	16.39±0.12b	99±0.58a

Values are replicates ± standard error. Significant differences at $p < 0.001$ were observed among cultivars in both germinability and average 1000 seed weight. Different letters in the columns indicate significant difference between cultivars at $\alpha = 0.05$ with Duncan's mean separation procedure.

Table S2. The arrangement of the treatment combinations and abbreviations used

Treatments	Treatment combinations	Abbreviations
T1	Asia green 1 control	G1A
T2	Asia green 1+0.5-mM MeJA	G1B
T3	Asia green 1+1.0-mM MeJA	G1C
T4	Asia green 2 control	G2A
T5	Asia green 2+0.5-mM MeJA	G2B
T6	Asia green 2+1.0-mM MeJA	G2C
T7	Asia red control	ARA
T8	Asia red+0.5-mM MeJA	ARB
T9	Asia red+1.0-mM MeJA	ARC
T10	Koregon red control	KRA
T11	Koregon red+0.5-mM MeJA	KRB
T12	Koregon red+1.0-mM MeJA	KRC
T13	Asia purple control	APA
T14	Asia purple+0.5-mM MeJA	APB
T15	Asia purple+1.0-mM MeJA	APC

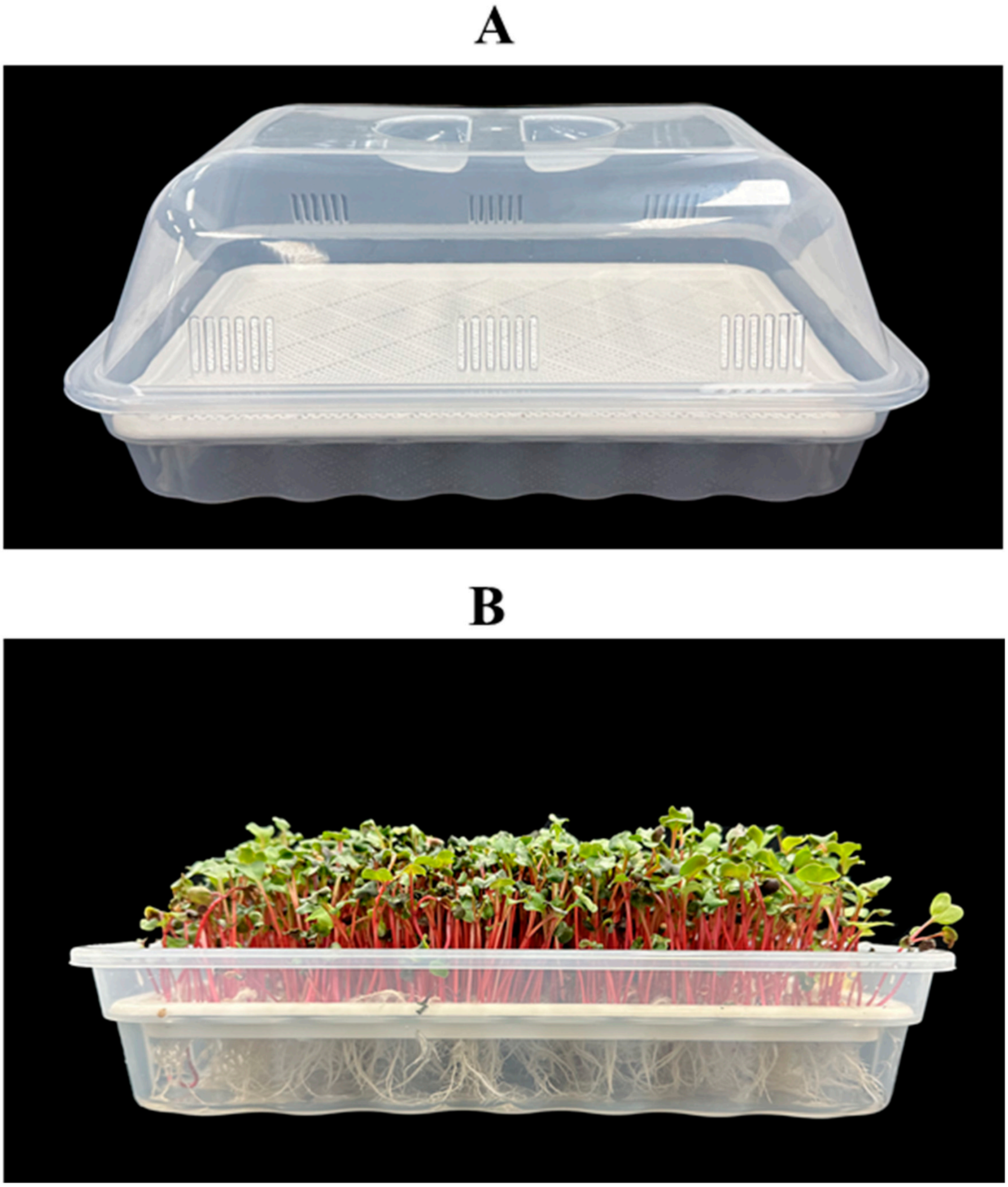

Figure S1. Growing tray (A) and harvesting stage (B) of radish microgreens.

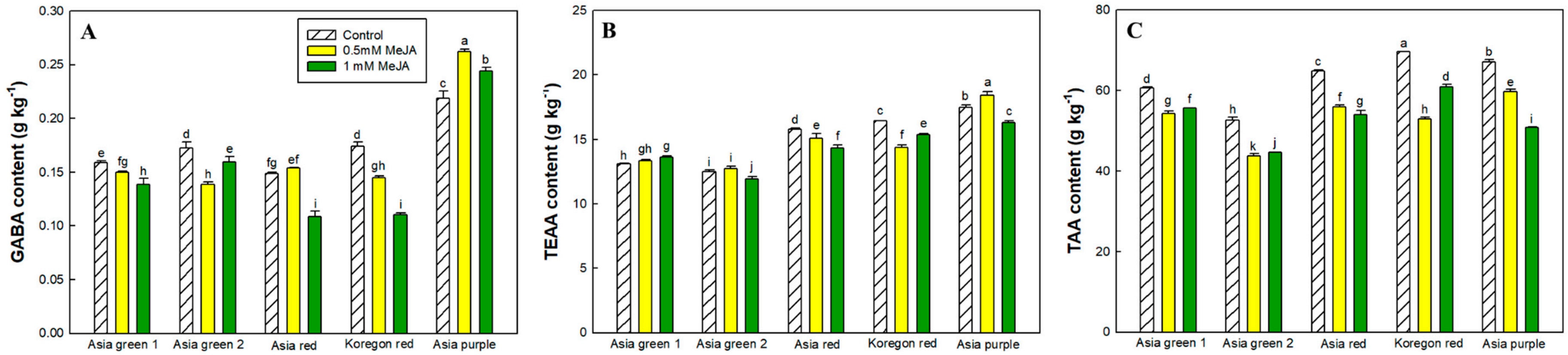

Figure S2. γ-aminobutyric acid (GABA) (A), total essential amino acids (B), and total amino acids (C) of five radish microgreen cultivars, cultivated without substrate and harvested on the 10th day, influenced by the interaction between the cultivars and preharvest MeJA treatment at 0.5 mM and 1.0 mM concentrations applied on the 7th day post-sowing. Vertical bars indicate average values of three replicates ± standard error. Different letters on the bars indicate significant differences between cultivars at $\alpha = 0.05$ with Duncan's mean separation procedure.

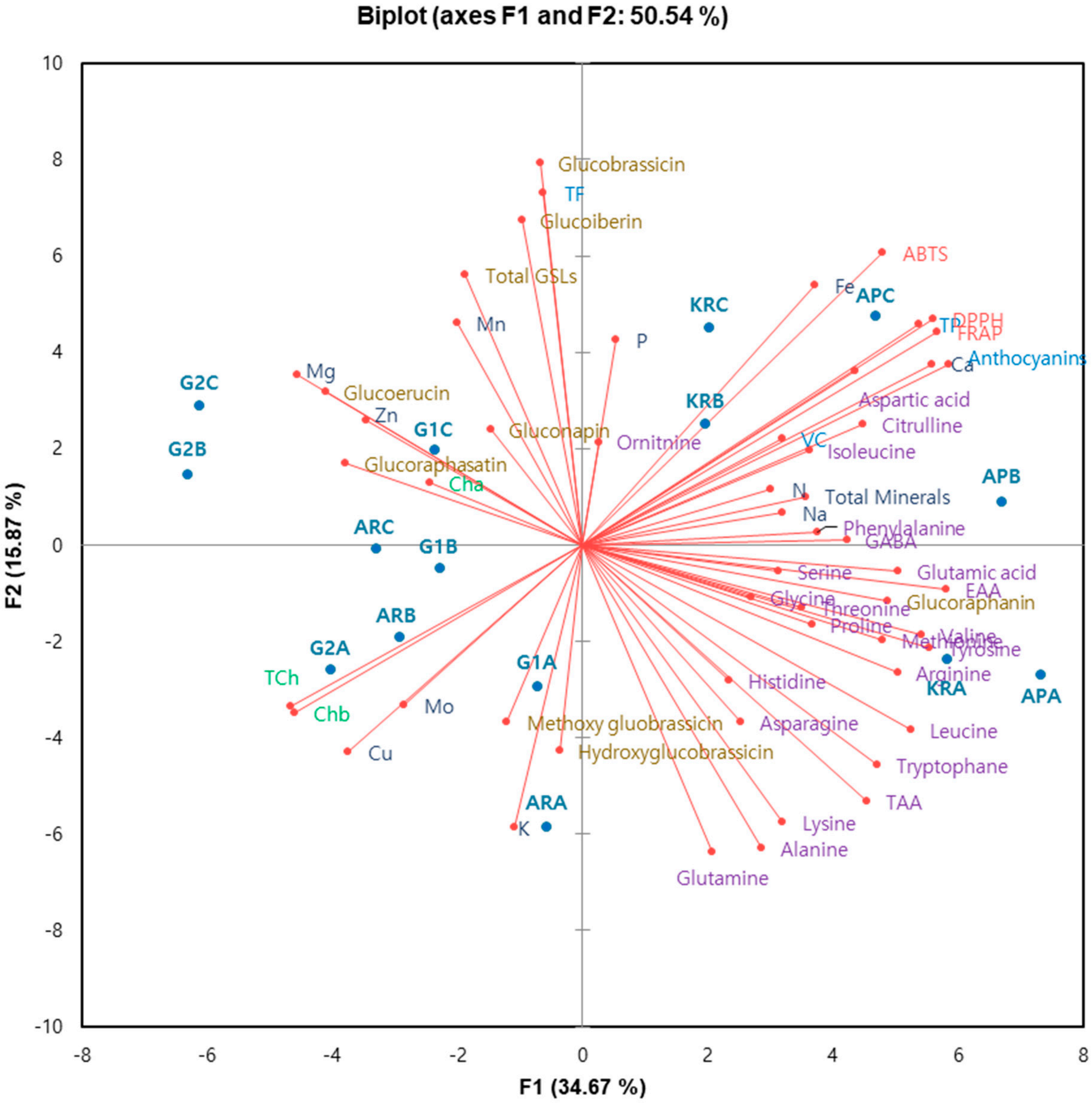

Figure S3. Biplot of five radish microgreen cultivars, cultivated without substrate and harvested on the 10th day, influenced by the interaction between the cultivars and preharvest MeJA treatment at 0.5 mM and 1.0 mM concentrations applied on the 7th day post-sowing. The data normalization was done by median combined with autoscaling, and these analyses were performed using MetaboAnalyst 5.0 software (https://www.metaboanalyst.ca/). G1, G2, AR, AP, KR, A, B, C, Cha, Chb, TCh, TAA, TEA, VC, TP, TF, Total GSLs, DPPH, FRAP, and ABTS represent ‘Asia green 1’, ‘Asia green 2’, ‘Asia red’, ‘Asia purple’, ‘Koregon red’, treatment A (control), treatment B (0.5-mM MeJA), treatment C (1.0-mM MeJA), chlorophyll a, chlorophyll b, total chlorophyll, total amino acids, total essential amino acids, vitamin C, total phenolics, total flavonoids, total glucosinolates, α-diphenyl-β-picrylhydrazyl, ferric reducing antioxidant power, and 2,2′-azino-bis (3-ethylbenzothiazoline-6-sulfonic acid), respectively.

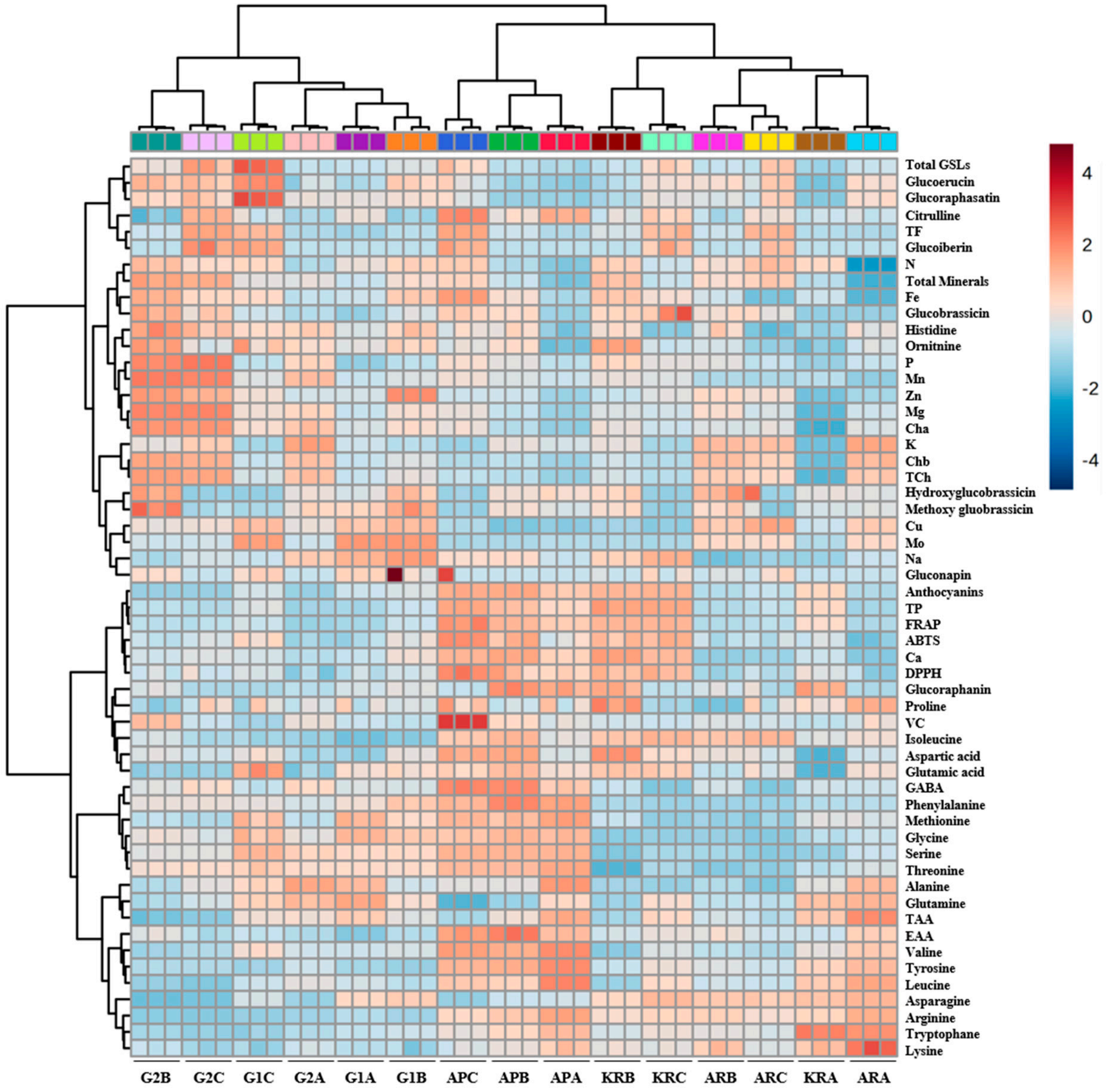

Figure S4. Heat map of five radish microgreen cultivars, cultivated without substrate and harvested on the 10th day, influenced by the interaction between the cultivars and preharvest MeJA treatment at 0.5 mM and 1.0 mM concentrations applied on the 7th day post-sowing. The data normalization was done by median combined with autoscaling, and the analysis was performed using MetaboAnalyst 5.0 software (https://www.metaboanalyst.ca/). G1, G2, AR, AP, KR, A, B, C, Cha, Chb, TCh, TAA, TEA, VC, TP, TF, Total GSLs, DPPH, FRAP, and ABTS represent 'Asia green 1', 'Asia green 2', 'Asia red', 'Asia purple', 'Koregon red', treatment A (control), treatment B (0.5-mM MeJA), treatment C (1.0-mM MeJA), chlorophyll a, chlorophyll b, total chlorophyll, total amino acids, total essential amino acids, vitamin C, total phenolics, total flavonoids, total glucosinolates, α-diphenyl-β-picrylhydrazyl, ferric reducing antioxidant power, and 2,2′-azino-bis (3-ethylbenzothiazoline-6-sulfonic acid), respectively.

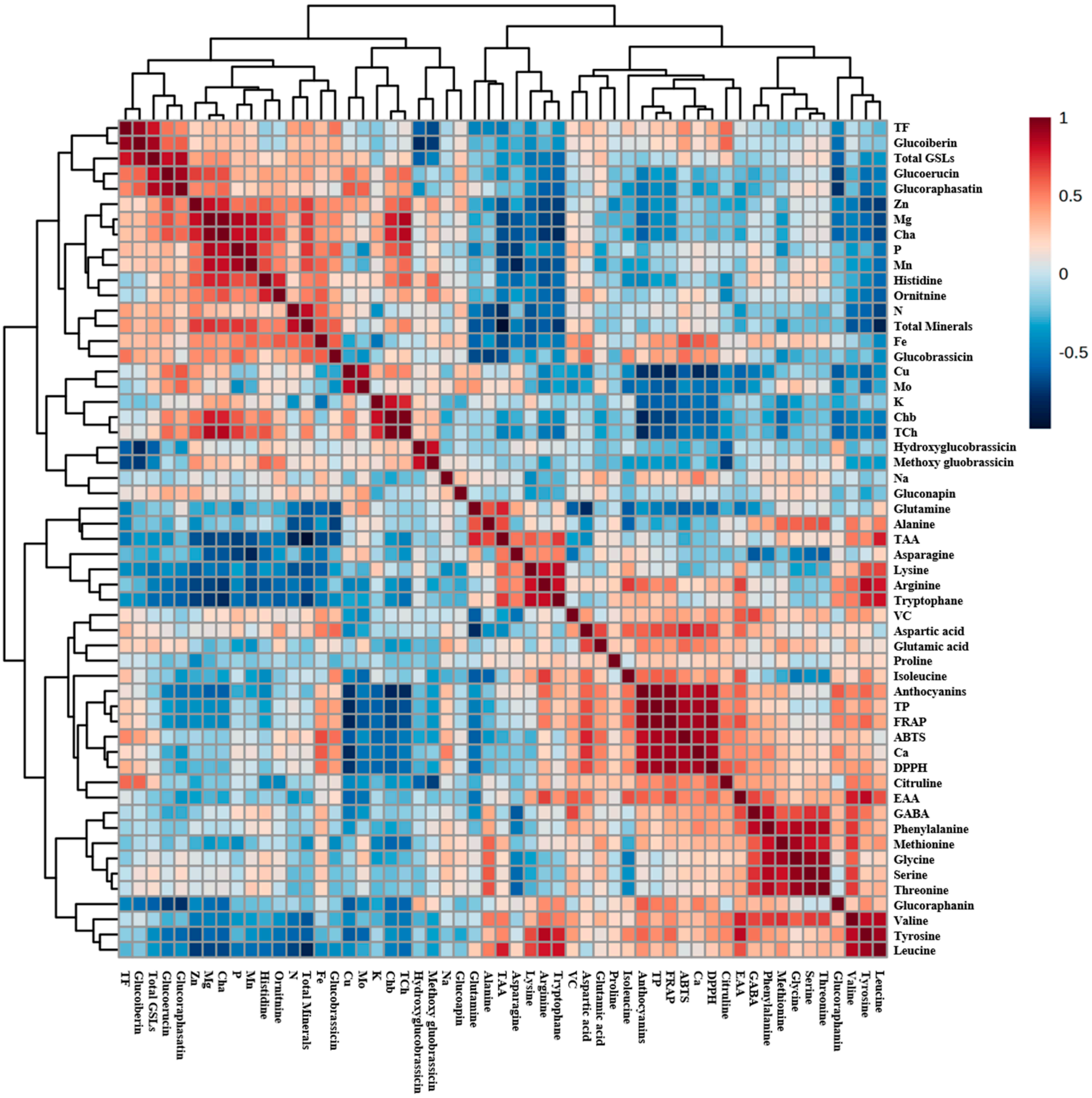

Figure S5. Correlation heat map of nutritional quality parameters in five radish microgreen cultivars, cultivated without substrate and harvested on the 10th day, influenced by the interaction between the cultivars and preharvest MeJA treatment at 0.5 mM and 1.0 mM concentrations applied on the 7th day post-sowing. Cha, Chb, TCh, TAA, TEA, VC, TP, TF, Total GSLs, DPPH, FRAP, and ABTS represent chlorophyll a, chlorophyll b, total chlorophyll, total amino acids, total essential amino acids, vitamin C, total phenolics, total flavonoids, total glucosinolates, α-diphenyl-β-picrylhydrazyl, ferric reducing antioxidant power, and 2,2′-azino-bis (3-ethylbenzothiazoline-6-sulfonic acid), respectively.
